# RIPK1 Drives JAK1‐STAT3 Signaling to Promote CXCL1‐Mediated Neutrophil Recruitment in Sepsis‐Induced Lung Injury

**DOI:** 10.1002/advs.202507123

**Published:** 2025-09-15

**Authors:** Hao Sun, Xiya Li, Shenjia Gao, Yi Jiang, Xinyi Wu, Zhaoyuan Chen, Han Wu, Qing Hua, Rui Zu, Huaijiang Xiang, Ronen Ben‐Ami, Changhong Miao, Li Tan, Ying Li, Jun Wang, Wankun Chen

**Affiliations:** ^1^ Department of Anesthesiology Zhongshan Hospital Fudan University Shanghai 200032 China; ^2^ Shanghai Key laboratory of Perioperative Stress and Protection Shanghai 200032 China; ^3^ Interdisciplinary Research Center on Biology and Chemistry Shanghai Institute of Organic Chemistry Chinese Academy of Sciences Shanghai 201210 China; ^4^ Infectious Diseases Unit Tel Aviv Sourasky Medical Center Faculty of Medicine Tel Aviv University Tel Aviv 6997801 Israel; ^5^ Department of Integrative Medicine and Neurobiology School of Basic Medical Science State Key Laboratory of Medical Neurobiology Fudan University Shanghai 200032 China; ^6^ Department of Anesthesiology Shanghai Geriatric Medical Center Shanghai 201104 China; ^7^ Department of Anesthesiology QingPu Branch of Zhongshan Hospital Affiliated to Fudan University Shanghai 201700 China

**Keywords:** alveolar epithelial cells, CXCL1, JAK1‐STAT3, signaling, neutrophil, RIPK1, sepsis‐induced lung injury

## Abstract

Sepsis‐induced lung injury, characterized by unregulated inflammation and impaired alveolar epithelial integrity, significantly contributes to sepsis‐related mortality. Although receptor‐interacting serine/threonine‐protein kinase 1 (RIPK1) is critical in regulating necroptosis and inflammation, its precise contribution to sepsis‐induced lung injury remains poorly understood. In this study, selective activation of RIPK1 in type II alveolar epithelial cells (AECs) is observed during sepsis. CXCL1 is identified as a critical downstream target of RIPK1 through integrative transcriptomic and proteomic analyses. Mechanistically, RIPK1 interacts with JAK1 to induce STAT3 phosphorylation, facilitate its nuclear translocation, and promote its binding to the *Cxcl1* promoter, thereby upregulating its expression and driving excessive neutrophil recruitment. Genetic or pharmacological inhibition of RIPK1 attenuated CXCL1 production, neutrophil infiltration, and alveolar damage, improving survival in septic mice. Compound 62, a selective RIPK1 inhibitor, has demonstrated efficacy in attenuating systemic inflammatory cascades, preserving epithelial barrier integrity, and improving survival rates in mice. These findings establish RIPK1 as a therapeutic target in sepsis‐induced lung injury and redefine alveolar epithelial cells as positive contributors to inflammatory amplification. This work advances precision strategies to mitigate sepsis‐induced lung injury, addressing a critical unmet need in critical care medicine.

## Graphical abstract

This article examines the central role of RIPK1 in the pathogenesis of sepsis‐induced lung injury. It elucidates how RIPK1 initiates an inflammatory cascade by activating JAK1‐STAT3 signaling, leading to CXCL1‐mediated neutrophil infiltration. Importantly, it demonstrates that pharmacological inhibition of RIPK1 effectively attenuates inflammation and improves lung outcomes. Figure generated with BioRender (www.biorender.com).

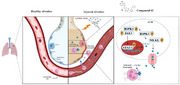

## 1 Introduction

Sepsis‐induced acute lung injury, a life‐threatening complication of systemic infection, remains a leading cause of mortality in critically ill patients, with mortality rates exceeding 40% in severe cases.^[^ ^1^, ^2^, ^3^, ^4^ ^]^ This condition is characterized by dysregulated inflammation, neutrophil‐driven tissue damage, and loss of alveolar epithelial integrity, culminating in respiratory failure.^[^ ^5^, ^6^ ^]^ While essential for pathogen clearance, neutrophils exacerbate injury when their recruitment and activation become excessive, releasing reactive oxygen species (ROS) and proteases that disrupt the alveolar‐capillary barrier.^[^ ^6^, ^7^, ^8^ ^]^ Chemokines such as CXCL1 play a pivotal role in neutrophil mobilization by binding to CXCR2, a process implicated in bacterial pneumonia and sepsis.^[^ ^9^, ^10^ ^]^ However, the cellular sources and upstream regulators of CXCL1 in sepsis‐induced lung injury remain incompletely understood.

Receptor‐interacting serine/threonine‐protein kinase 1 (RIPK1), recognized for its role in necroptosis via RIPK3‐MLKL signaling, has emerged as a critical mediator of inflammation.^[^ ^11^, ^12^, ^13^ ^]^ Recent clinical studies report elevated RIPK1 activity in patients with severe infections, including COVID‐19, correlating with organ dysfunction.^[^ ^14^ ^]^ Preclinical models further implicate RIPK1 as a key regulator of cytokine storms and endothelial apoptosis,^[^ ^15^, ^16^ ^]^ highlighting its role in amplifying systemic inflammatory responses and contributing to vascular dysfunction in sepsis‐induced organ injury. However, its cell type‐specific contributions in the lung, particularly within structural cells like alveolar epithelial cells (AECs), are poorly defined. Most research has focused on immune cells, such as macrophages and neutrophils, as primary drivers of chemokine production.^[^ ^17^, ^18^ ^]^ Yet emerging evidence suggests that AECs—traditionally viewed as passive targets—actively orchestrate pulmonary inflammation through chemokine secretion and direct cross‐talk with immune cells.^[^ ^19^, ^20^, ^21^ ^]^ However, the role of RIPK1 in regulating AEC‐driven inflammatory amplification remains unexplored. This gap is critical because AECs are uniquely positioned to initiate localized inflammatory cascades. As first‐line responders to pathogens and mediators of alveolar‐capillary barrier integrity, their role is distinct from that of migratory immune cells.

Emerging evidence suggests that RIPK1 may regulate inflammatory signaling independently of necroptosis. For instance, RIPK1 kinase activity can modulate NF‐κB and MAPK pathways, influencing cytokine production.^[^ ^12^, ^22^ ^]^ Intriguingly, the JAK1‐STAT3 axis, a canonical pathway activated by cytokines like IL‐6, has been linked to CXCL1 transcription in cancers.^[^ ^23^, ^24^ ^]^ Yet, whether RIPK1 interacts with JAK1‐STAT3 to drive chemokine expression in AECs remains unexplored.

While RIPK1 inhibition has emerged as a promising therapeutic strategy for inflammatory diseases, clinical translation of existing inhibitors remains challenging. Preclinical studies with first‐generation inhibitors like Necrostatin‐1 showed survival benefits in sepsis models by suppressing necroptosis and cytokine release.^[^ ^25^, ^26^ ^]^ However, their clinical utility is hindered by off‐target effects, metabolic instability, and suboptimal pharmacokinetics.^[^ ^27^, ^28^ ^]^ Critically, no RIPK1 inhibitor has yet achieved clinical efficacy in sepsis‐induced lung injury, underscoring the unmet need for more selectivity and pharmacokinetically optimized agents. To bridge this translational gap, we leveraged compound 62 (C62), a novel type II kinase inhibitor exhibiting sub‐nanomolar potency against both murine and human RIPK1, coupled with favorable oral bioavailability and extended half‐life.^[^ ^29^ ^]^

In this study, we hypothesize that RIPK1 activation in type II alveolar epithelial cells (ATII) promotes CXCL1‐driven neutrophil recruitment via JAK1‐STAT3 signaling, independently of necroptosis, thereby exacerbating sepsis‐induced lung injury. Using genetic and pharmacological approaches in murine models, we aim to delineate the spatiotemporal activation of RIPK1 in AECs during sepsis, elucidate its mechanistic link to JAK1‐STAT3‐mediated CXCL1 transcription, and evaluate the efficacy of a novel RIPK1 inhibitor, compound 62,^[^ ^29^ ^]^ in mitigating lung injury. Our findings challenge the immune cell‐centric paradigm of neutrophilic inflammation, repositioning AECs as active contributors to pathological signaling. By identifying a RIPK1‐JAK1‐STAT3‐CXCL1 axis, this work not only expands RIPK1's non‐necroptotic functions but also provides a precision‐targeted therapeutic strategy to improve outcomes in sepsis‐induced ALI.

## 2 Results

### 2.1 Significant Activation of RIPK1 in Alveolar Epithelial Cells During Sepsis‐Induced Lung Injury

**Figure 1:**
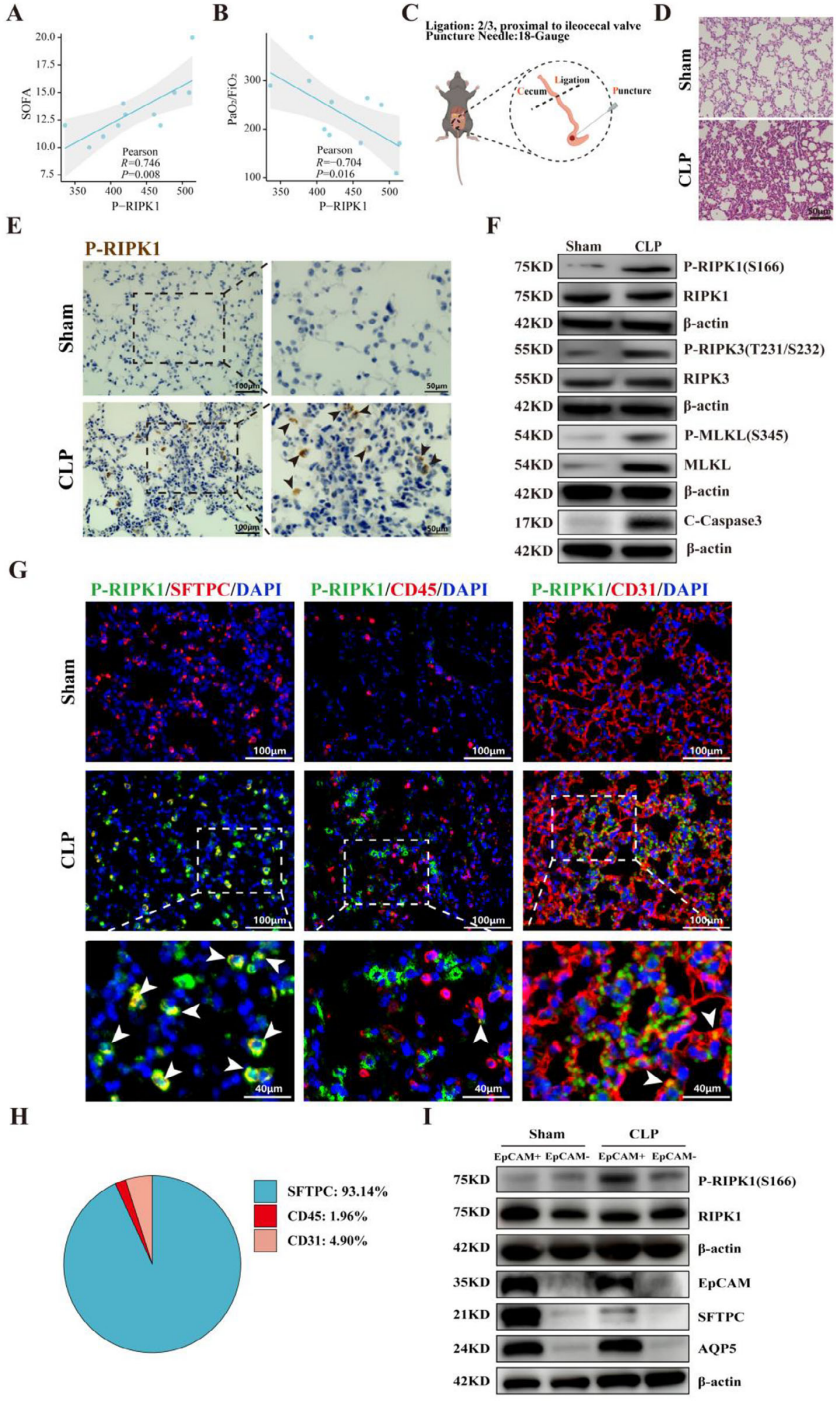
Significant Activation of RIPK1 in Alveolar Epithelial Cells During Sepsis‐Induced Lung Injury A,B) Correlation analyses between phosphorylated RIPK1 (P‐RIPK1) levels and clinical parameters, including Sequential Organ Failure Assessment (SOFA) scores (A) and PaO_2_/FiO_2_ ratios (B), in sepsis‐induced lung injury. Pearson's correlation coefficients and P‐values are shown (n = 11). C) Schematic representation of the cecal ligation and puncture (CLP) model used to induce sepsis in mice. The cecum was ligated at two‐thirds of its length proximal to the ileocecal valve and punctured with an 18‐gauge needle. D) Representative hematoxylin and eosin (H&E) staining of lung tissue from sham and CLP groups, showing inflammatory infiltration and alveolar damage in the CLP group. Scale bar: 50µm. E) Immunohistochemical staining for P‐RIPK1 in lung tissue from sham and CLP groups. Positive signals are indicated by brown staining. Arrowheads indicate P‐RIPK1‐positive cells. Scale bar: 100 and 50µm. F) Western blot analysis of P‐RIPK1(S166), RIPK1, P‐RIPK3 (T231/S232), RIPK3, P‐MLKL (S345), MLKL, and cleaved Caspase‐3 (C‐Caspase‐3) in lung tissues from sham and CLP groups. β‐actin served as a loading control (n = 4). G) Immunofluorescence staining of lung tissue showing co‐localization of P‐RIPK1 (green) with different cell markers: SFTPC (alveolar epithelial type II cells, red), CD45 (leukocytes, red), and CD31 (endothelial cells, red). Nuclei are stained with DAPI (blue). Arrowheads indicate co‐localization. Scale bars: 100 and 40µm. H) Quantification of P‐RIPK1‐positive cells in lung tissue, categorized based on marker expression: SFTPC (93.14%), CD45 (1.96%), and CD31 (4.90%). I) Western blot analysis of epithelial cell markers (EpCAM, SFTPC, and AQP5) and P‐RIPK1 levels in EpCAM‐positive and EpCAM‐negative fractions from sham and CLP groups. β‐actin served as a loading control.

Studies have shown that RIPK1, a necroptosis‐associated protein, is continuously elevated in the plasma of patients with severe COVID‐19 driven by cytokine storms.^[^ ^14^ ^]^ Our clinical analysis of sepsis patients demonstrated that plasma P‐RIPK1 levels positively correlated with Sequential Organ Failure Assessment (SOFA) scores (**Figure** **1**A) and inversely correlated with the PaO_2_/FiO_2_ ratio (Figure 1B), suggesting its prognostic utility for systemic organ dysfunction and hypoxemic respiratory failure. These data indicate that P‐RIPK1 as a biomarker of sepsis's disease severity and lung injury progression.

To investigate RIPK1 activation dynamics in sepsis‐induced lung injury, we employed a lethal cecal ligation and puncture (CLP) model in adult male mice, in which survival is limited to a maximum of 72 h (Figure 1C; Figure S1A, Supporting Information). Histopathological analysis revealed multi‐organ injury, including pulmonary edema, hepatic necrosis, and renal tubular damage, at 24 h post‐CLP (Figure 1D; Figure S1B, Supporting Information). Immunohistochemistry and western blot analysis revealed marked upregulation of phosphorylated RIPK1 (Figure S1, Supporting Information) in septic lungs (Figure 1E,F; Figure S1C, Supporting Information), consistent with activation of its downstream effectors P‐RIPK3 (T231/ Figure S2, Supporting Information), P‐MLKL (Figure S3, Supporting Information), and cleaved Caspase‐3 (Figure 1F; Figure S1C, Supporting Information). These findings establish RIPK1 as a molecular switch that regulates cell death in sepsis by integrating inflammatory mediators and cellular stress cues.^[^ ^11^, ^13^ ^]^ In line with human patient data, we observed a significant positive correlation between serum P‐RIPK1 levels and established murine sepsis scores (MSS)^[^ ^30^, ^31^ ^]^ in our murine CLP model (Figure S1D, Supporting Information), supporting its role as a conserved biomarker for sepsis severity across species. To delineate the causal relationship between RIPK1 activation and lung injury, we performed a time‐course analysis in the CLP model. Phosphorylation of RIPK1 (Figure S1, Supporting Information) was significantly elevated in lung homogenates at 3 h post‐CLP (Figure S1E, Supporting Information), whereas histopathological signs of lung injury (alveolar wall thickening, hemorrhage, and neutrophil infiltration) appeared at 6 h (Figure S1F, Supporting Information). This temporal sequence—RIPK1 activation preceding tissue damage—establishes RIPK1 as an upstream driver of pathological signaling in sepsis‐induced lung injury.

To specifically map the cellular sites of RIPK1 activation during sepsis pathogenesis, we performed multiplex immunofluorescence staining on lung sections from CLP model mice. Co‐staining with lineage markers demonstrated striking spatial compartmentalization: P‐RIPK1 predominantly colocalized with SFTPC+ alveolar type II (ATII) epithelial cells, while showing minimal association with CD45+ leukocytes or CD31+ endothelial cells (Figure 1G,H). This distinct epithelial activation pattern prompted us to investigate potential RIPK1‐mediated dysfunction in ATII cells. Using EpCAM‐specific magnetic sorting for pulmonary epithelial isolation (Figure S1G, Supporting Information), we performed compartmentalized protein analysis. Western blot quantification demonstrated significant enrichment of P‐RIPK1 in EpCAM+ fractions compared to their EpCAM‐ counterparts (Figure 1I). Notably, this RIPK1 activation signature correlated with selective depletion of the ATII‐specific marker SFTPC, while maintaining stable expression of the ATI marker AQP5 (Figure 1I), suggesting differential susceptibility between alveolar epithelial subtypes.

To establish an ex vivo model of RIPK1‐dependent signaling, we stimulated MLE‐12 alveolar epithelial cells with LPS (1µgmL^−1^, 24 h), a pathogen‐associated molecular pattern (PAMP) implicated in sepsis pathogenesis.^[^ ^32^, ^33^, ^34^ ^]^ This treatment induced significant RIPK1/RIPK3/MLKL activation (Figure S1H, Supporting Information), confirming LPS as a potent driver of RIPK1‐mediated inflammatory cascades in pulmonary epithelium.

### 2.2 Systemic Inhibition of RIPK1 Kinase Activity Significantly Alleviates Septic Lung Injury

**Figure 2:**
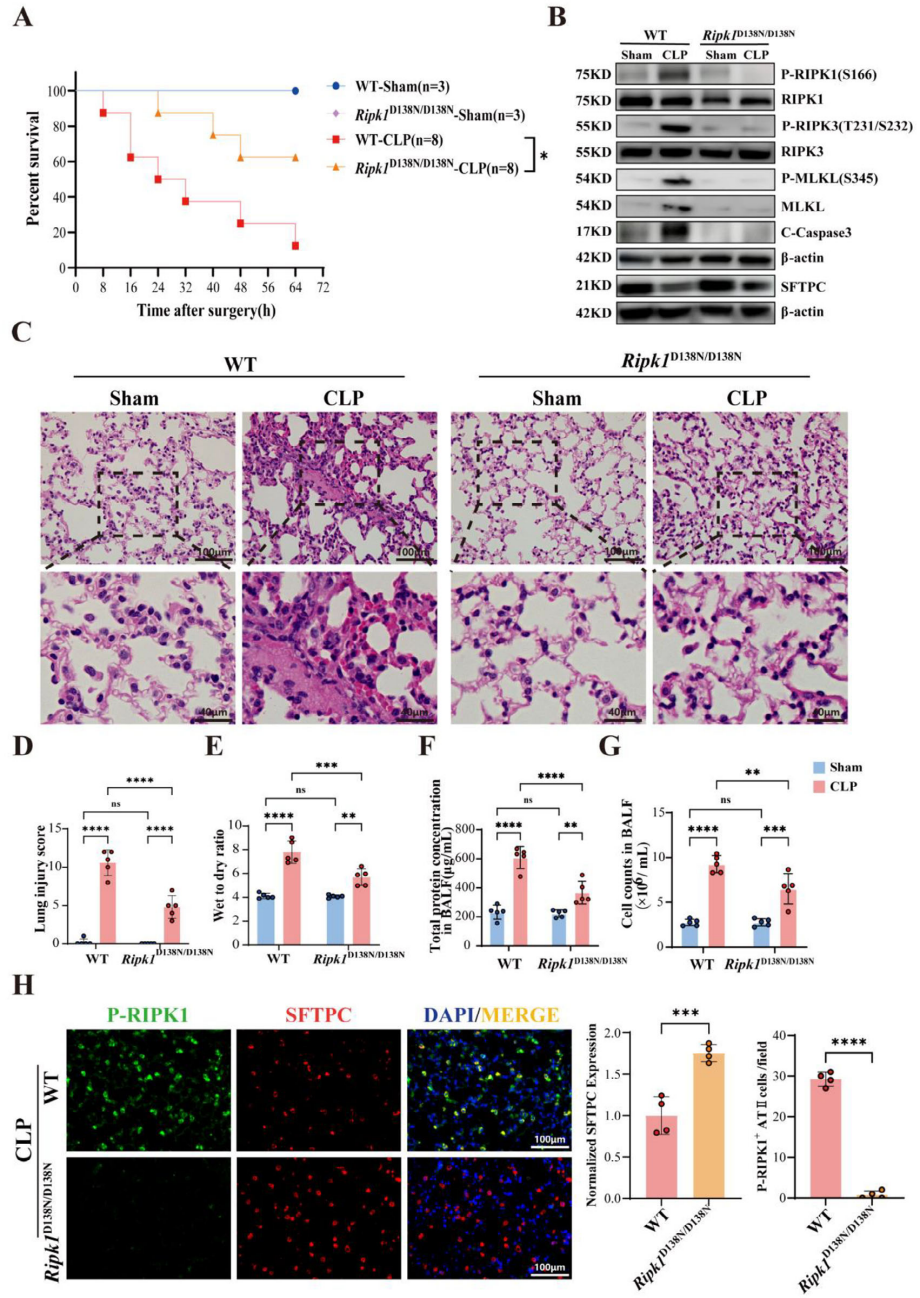
Systemic Inhibition of RIPK1 Kinase Activity Significantly Alleviates Septic Lung Injury. A) Kaplan‐Meier survival curves showing the percentage survival of WT and *Ripk1* ^D138N/D138N^ mice in sham and CLP groups over 72 h (Sham groups, n = 3; CLP groups, n = 8). B) Western blot analysis of P‐RIPK1(S166), RIPK1, P‐RIPK3(T231/S232), RIPK3, P‐MLKL(S345), MLKL, Cleaved Caspase‐3 (C‐Caspase3), and SFTPC levels in lung tissues of WT and *Ripk1* ^D138N/D138N^ mice after sham or CLP surgery. β‐actin served as a loading control. C) Representative H&E‐stained lung tissue sections from WT and *Ripk1* ^D138N/D138N^ mice in sham and CLP groups, showing tissue morphology, inflammatory infiltration, and alveolar damage. Scale bar: 100 and 40µm. D–G) Quantification of lung injury parameters: D) Lung injury scores, E) lung wet/dry weight ratio, F) total protein concentration in bronchoalveolar lavage fluid (BALF), and G) cell counts in BALF from WT and *Ripk1* ^D138N/D138N^ mice (n = 5). H) Immunofluorescence staining of lung tissue showing P‐RIPK1 (green) and SFTPC (alveolar epithelial type II cell marker, red) in WT and *Ripk1* ^D138N/D138N^ mice under CLP condition. Nuclei were stained with DAPI (blue). Scale bars: 100µm. Quantification of SFTPC expression and P‐RIPK1‐positive alveolar epithelial type II cells per lung tissue area is shown on the right (n = 4). Data are presented as mean±SD. Survival comparisons were analyzed using the log‐rank test (A) Multiple comparisons were analyzed using two‐way ANOVA (D–G). Two‐group comparisons were analyzed using Student's *t*‐test (H). ****P < 0.0001;***P < 0.001; **P < 0.01; *P < 0.05; NS, no significant difference. SD, standard deviation; ANOVA, analysis of variance.

To further explore the role of RIPK1 kinase activity in the development of sepsis, we used *Ripk1* ^D138N/D138N^ mice, which harbor a kinase‐dead mutation,^[^ ^15^, ^35^, ^36^ ^]^ in the CLP model. Notably, *Ripk1* ^D138N/D138N^ mice showed significantly higher survival rates than their wild‐type (WT) counterparts after CLP surgery (**Figure** **2**A), concurrent with abolished RIPK1 autophosphorylation (Figure S1, Supporting Information) and downstream RIPK3/MLKL/Caspase‐3 activation in septic lungs (Figure 2B). Histopathological analysis of HE‐stained sections revealed attenuated alveolar damage in mutant mice, including suppressed wall thickening, edema, hyaline membrane formation, and inflammatory infiltration, with correspondingly reduced lung injury scores (Figure 2C,D). Consistent with improved survival and these histopathological findings, the mutant mice demonstrated reduced alveolar‐capillary barrier disruption as evidenced by diminished bronchoalveolar lavage fluid (BALF) protein/cellularity and lung wet‐to‐dry ratio (Figure 2E–G). Crucially, *Ripk1* ^D138N/D138N^ mice preserved type II alveolar epithelial cell integrity (Figure 2B,H), directly implicating RIPK1 kinase activity in driving ATII cell loss and barrier dysfunction during sepsis. Taken together, these findings provide robust evidence that the inhibition of RIPK1 kinase activity substantially mitigates acute pulmonary pathological alterations induced by sepsis.

### 2.3 Conditional Knockout of RIPK1 in Alveolar Epithelial Cells Mitigate Sepsis‐Induced Lung Injury

**Figure 3:**
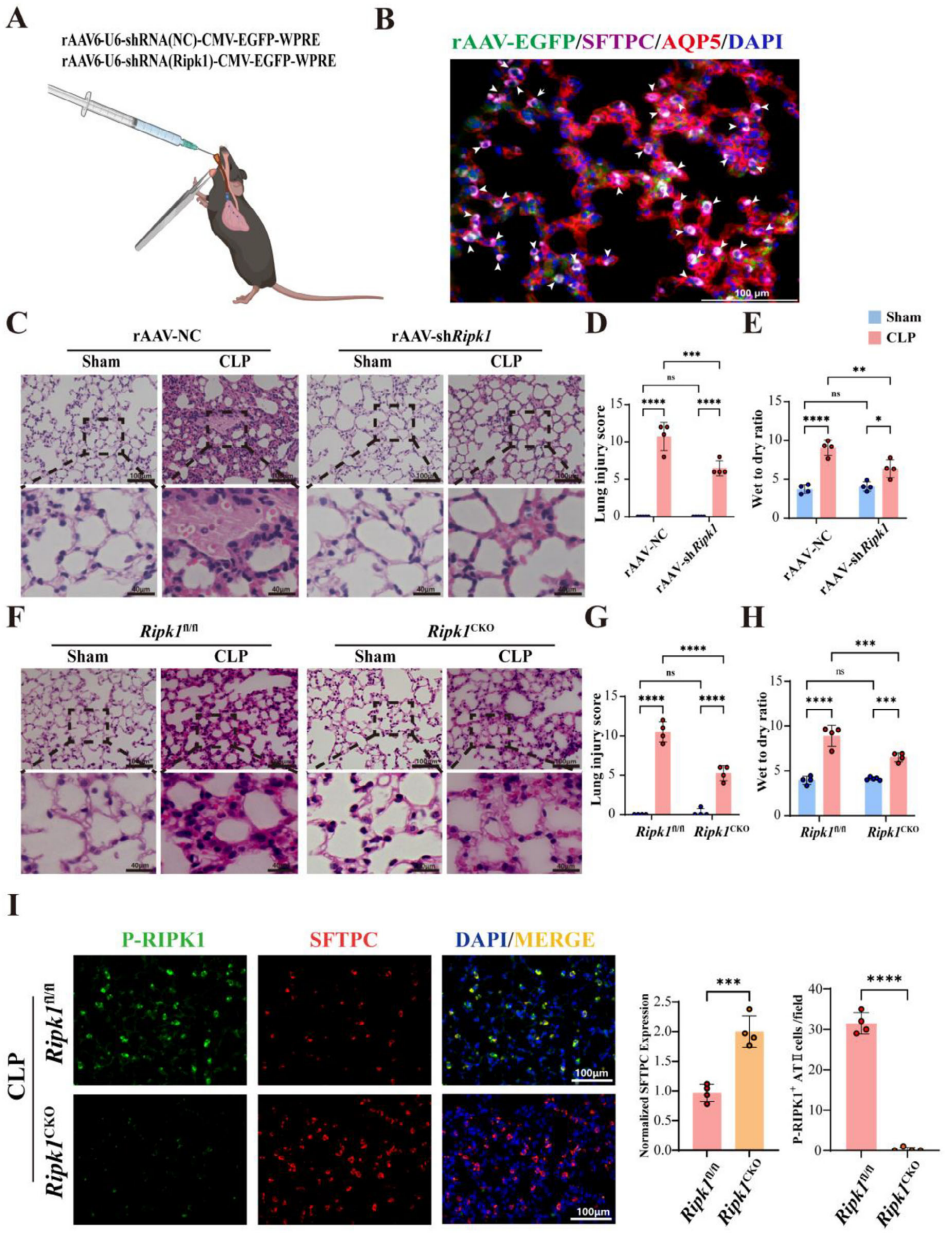
Conditional Knockout of RIPK1 in Alveolar Epithelial Cells Mitigates Sepsis‐Induced Lung Injury. A) Schematic illustration of the intratracheal administration of recombinant adeno‐associated virus (rAAV) carrying shRNA targeting *Ripk1* or control shRNA. B) Representative immunofluorescence images of rAAV‐EGFP (green), SFTPC (purple), and AQP5 (red) in lung sections. Nuclei were counterstained with DAPI (blue). Arrowheads indicate cells coexpressing P‐RIPK1 and SFTPC. Scale bar: 100µm. C) Representative H&E‐stained lung sections from rAAV‐NC and rAAV‐sh*Ripk1* groups in sham and CLP conditions. Scale bar : 100 and 40µm. D–E) Quantification of lung injury scores D) and wet‐to‐dry lung weight ratios E) in rAAV‐NC and rAAV‐sh*Ripk1* groups following CLP‐induced injury (n = 4). F) Representative H&E‐stained lung sections from *Ripk1* ^fl/fl^ and *Ripk1* ^CKO^ mice in sham and CLP conditions. Scale bar: 100 and 40µm. G–H) Quantification of lung injury scores (G) and wet‐to‐dry ratios (H) in *Ripk1* ^fl/fl^ and *Ripk1* ^CKO^ mice following CLP (n = 4). I) Immunofluorescence staining of P‐RIPK1 (green), SFTPC (red), and DAPI (blue) in lung tissues from *Ripk1* ^fl/fl^ and *Ripk1* ^CKO^ mice under CLP condition. Scale bar: 100µm (n = 4). Quantification of SFTPC expression and P‐RIPK1‐positive alveolar epithelial type II cells per lung tissue area is shown on the right. Data are presented as mean ± SD. Multiple comparisons were analyzed using two‐way ANOVA (D‐E, G‐H). Two‐group comparisons were analyzed using Student's *t*‐test (I). ****P < 0.0001;***P < 0.001; **P < 0.01; *P < 0.05; NS, no significant difference; SD, standard deviation. ANOVA, analysis of variance.

To further explore the role of alveolar epithelial cell RIPK1 in sepsis‐induced lung injury, we carried out a gene knockdown experiment using a recombinant adeno‐associated virus (rAAV) system delivered through intratracheal instillation. Leveraging the pulmonary tropism of the AAV6 serotype and the high transcriptional efficiency of the U6 promoter, we constructed a targeted gene‐silencing system for alveolar epithelial cells. The AAV6 serotype was selected due to its specific binding to heparan sulfate proteoglycans (HSPGs) in the pulmonary basement membrane,^[^ ^37^, ^38^ ^]^ demonstrating significantly higher transduction efficiency in alveolar epithelial cells compared to other serotypes (e.g., AAV2).^[^ ^39^, ^40^, ^41^ ^]^ The viral vector design incorporated a U6 promoter‐driven shRNA expression cassette, where this RNA polymerase III‐dependent promoter ensures sustained high‐level expression of small RNA molecules within target cells.^[^ ^42^ ^]^ In the experimental group, mice received rAAV6‐U6‐shRNA (*Ripk1*)‐CMV‐EGFP‐WPRE to specifically knockdown *Ripk1* in alveolar epithelial cells, while the control group received rAAV6‐U6‐shRNA (NC)‐CMV‐EGFP‐WPRE (**Figure** **3**A). Three weeks after instillation, immunofluorescence analysis confirmed that the virus was confined to the lung (Figure S2A, Supporting Information), with predominant infection of type II alveolar epithelial cells (Figure 3B). The knockdown efficiency of *Ripk1* in alveolar epithelial cells was verified, and RIPK1 expression in the knockdown group was significantly reduced compared to the control group (Figure S2B,C, Supporting Information). Our experiments demonstrated that knockdown of *Ripk1* in alveolar epithelial cells significantly mitigated sepsis‐induced lung pathology, including alveolar hemorrhage and inflammatory infiltrates (Figure 3C–E).

To address rAAV off‐target limitations, we utilized a conditional gene knockout approach to further clarify the essential role and pathogenic mechanism of RIPK1 in ATII cells during sepsis‐induced lung injury. By interbreeding *Sftpc*‐CreER/+ mice with *Ripk1* ^flox/flox^ mice, we successfully created a mouse model featuring ATII cell‐specific *Ripk1* knockout (*Ripk1* ^flox/flox^; *Sftpc*‐CreER/+) (Figure S2D‐F). To further evaluate the functional impact of epithelial RIPK1 ablation on survival, we monitored *Ripk1* ^CKO^ mice (*Ripk1* ^flox/flox^; *Sftpc*‐CreER/+) and *Ripk1* ^fl/fl^ littermate controls for 72 h post‐CLP. Genetic deletion of RIPK1 in AECs significantly improved survival rates (Figure S2G, Supporting Information). This survival benefit was associated with attenuated lung pathology (Figure 3F–H), including reduced alveolar hemorrhage and leukocyte infiltration, and preserved ATII cell populations (Figure 3I; Figure S2H, Supporting Information), underscoring the pivotal role of epithelial RIPK1 in driving lethal inflammation during sepsis.

Considering that RIPK1 plays a dual role in regulating cell survival and death,^[^ ^12^, ^13^ ^]^ we evaluated the safety profile of conditionally knocking out RIPK1 in alveolar epithelial cells. Neither pulmonary cellular apoptosis nor alveolar architecture showed significant perturbations following RIPK1 deletion (Figure 3F; Figure S2I, Supporting Information). Consistently, in vitro, knockdown models revealed preserved cellular viability without detectable cytopathic effects (Figure S2J–L, Supporting Information). Collectively, these findings indicate that RIPK1 may be dispensable for maintaining baseline apoptotic homeostasis in the pulmonary epithelium under normal physiological conditions.

Taken together, these results showed RIPK1 as a key driver of ATII cell injury and sepsis‐induced lung injury.

### 2.4 RIPK1 Mediates CXCL1‐Driven Neutrophil Recruitment in Sepsis‐Induced Lung Injury

**Figure 4:**
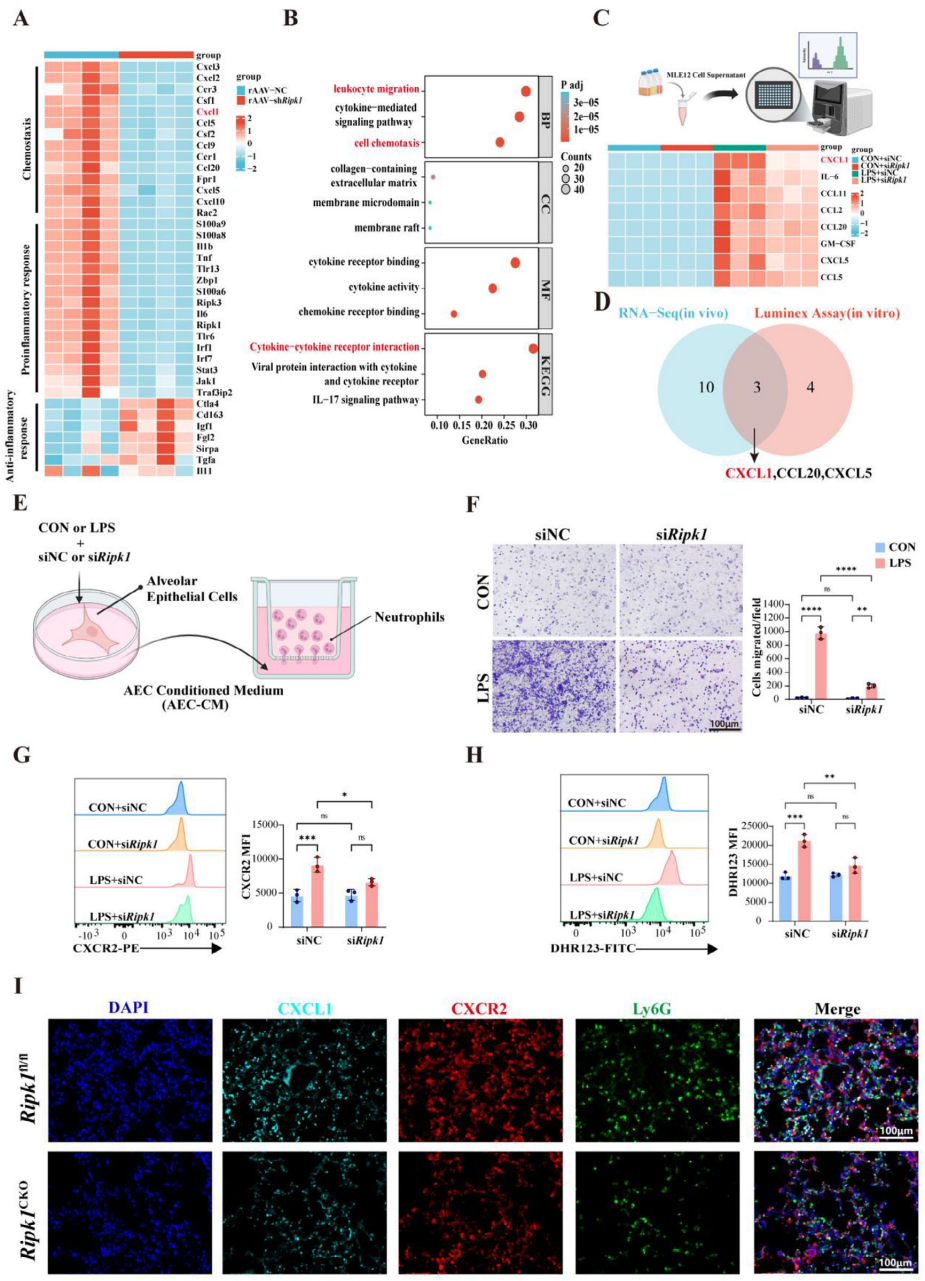
RIPK1 Mediates CXCL1‐Driven Neutrophil Recruitment in Sepsis‐Induced Lung Injury. A) Heatmap of chemokine and inflammatory response gene expression in AECs from rAAV‐NC and rAAV‐sh*Ripk1* mice under CLP treatment conditions, based on RNA‐seq analysis. B) Gene Ontology (GO) and Kyoto Encyclopedia of Genes and Genomes (KEGG) pathway enrichment analysis of differentially expressed genes. GO terms include biological processes (BP), cellular components (CC), and molecular functions (MF), highlighting pathways such as leukocyte migration, cytokine‐mediated signaling, and membrane microdomains. KEGG pathway analysis reveals enrichment in cytokine‐cytokine receptor interaction and IL‐17 signaling. C) Luminex multiplex array of cell culture supernatants from MLE12 alveolar epithelial cells treated with siNC or si*Ripk1* under CON or LPS conditions. Heatmap showing the relative expression of key chemokines (e.g., CXCL1, IL‐6, CCL11, CCL2, and CCL20) in different treatment groups. D) Venn diagram of RIPK1‐regulated chemokines screened by combined RNA‐seq (in vivo) and Luminex assay (in vitro), identifying CXCL1, CCL20, and CXCL5 as common targets. E) Schematic illustration of an in vitro neutrophil migration assay using conditioned medium (AEC‐CM) from alveolar epithelial cells transfected with siNC or siRipk1 and stimulated with CON or LPS. F) Representative images of neutrophil migration assays under different conditions (siNC or si*Ripk1* with CON or LPS stimulation). Quantification of migrated neutrophils per field is shown on the right. Scale bar: 100µm (n = 3). G) Flow cytometry analysis of CXCR2 expression in neutrophils exposed to AEC‐CM from different conditions. Quantification of CXCR2 mean fluorescence intensity (MFI) is shown (n = 3). H) Flow cytometry analysis of reactive oxygen species (ROS) production in neutrophils using DHR123 fluorescence, with quantification of mean fluorescence intensity (MFI) (n = 3). I) Representative immunofluorescence images of lung tissues from *Ripk1* ^fl/fl^ and *Ripk1* ^CKO^ mice under CLP condition, showing the expression of CXCL1 (cyan), CXCR2 (red), Ly6G (green), and nuclei (DAPI, blue). Scale bar: 100µm. Data are presented as mean ± SD. Multiple comparisons were analyzed using two‐way ANOVA (F–H). ****P < 0.0001;***P < 0.001; **P < 0.01; *P < 0.05; NS, no significant difference; ANOVA, analysis of variance.

To better investigate the mechanisms by which RIPK1 in alveolar epithelial cells regulates lung injury, we conducted transcriptomic sequencing on alveolar epithelial cells isolated from CLP mice treated with *Ripk1* knockdown rAAV and the negative control (NC) group. Unsupervised clustering analysis, including principal component analysis (PCA) and hierarchical clustering heatmaps (Figure S3A,B, Supporting Information), revealed 3060 differentially expressed genes (DEGs) between *Ripk1* knockdown and control groups, indicating substantial transcriptional reprogramming upon RIPK1 deficiency. To uncover functionally relevant biological processes, we performed rigorous functional annotation. Gene Ontology (GO) and Kyoto Encyclopedia of Genes and Genomes (KEGG) pathway enrichment analyses revealed significant enrichment of DEGs in categories critical for inflammatory cell recruitment, including “Cytokine‐cytokine receptor interaction”, “Leukocyte migration” and “Cell Chemotaxis” (**Figure** **4**A,B). This findings suggested that RIPK1 primarily regulates sepsis‐induced lung injury by modulating inflammatory cell recruitment. Chemokines play a pivotal role in sepsis‐induced lung injury by orchestrating excessive inflammation through immune cell recruitment and activation.^[^ ^43^ ^]^ These signaling molecules attract neutrophils and monocytes to lung tissue, where their uncontrolled activation disrupts the alveolar‐capillary barrier. The consequent pulmonary edema and tissue damage create a vicious cycle: chemokine‐mediated feedback loops perpetuate inflammation while simultaneously impairing immune regulation.^[^ ^5^ ^]^ This cascade culminates in a pathogenic “cytokine storm” that not only exacerbates local lung damage but also propagates systemic inflammation, potentially triggering multi‐organ failure—a hallmark of severe sepsis progression.^[^ ^2^, ^44^ ^]^ Given their well‐documented role in sepsis‐related lung damage, these findings strongly support further research into chemokine‐focused therapies.

Based on transcriptomic data suggesting that RIPK1 regulates chemotaxis, we employed siRNA‐mediated knockdown to validate its functional role in chemokine secretion. Efficient *Ripk1* silencing significantly attenuated LPS‐induced chemokine release in alveolar epithelial cells, as quantified by Luminex liquid suspension chip assay (Figure 4C; Figure S3C, Supporting Information). Integrative analysis of RNA‐Seq and Luminex Assay datasets identified three overlapping chemokines (CXCL1, CCL20, and CXCL5) at the transcriptomic‐proteomic interface (Figure 4D). Strikingly, CXCL1 exhibited the highest transcriptional upregulation in CLP models and correspondingly dominated protein secretion in supernatants. Furthermore, *Ripk1* knockdown attenuated CXCL1 expression more robustly than other chemokines (Figure 4A,C; Figure S3C, Supporting Information), establishing it as the central effector of RIPK1‐driven inflammatory cascades. To elucidate the primary cellular source of CXCL1 in sepsis‐induced lung injury, we performed multicolor immunofluorescence co‐staining analysis on lung tissue sections from CLP mice. Analysis showed extensive and significant co‐localization of CXCL1 with SFTPC, a marker for Type II alveolar epithelial cells (ATII). In contrast, co‐localization of CXCL1 with the macrophage marker F4/80 or the endothelial cell marker CD31 was very limited (Figure S3D,E, Supporting Information). These in vivo data strongly support our in vitro findings that alveolar epithelial cells are a major source of CXCL1 after LPS stimulation (Figure S3F, Supporting Information), positioning alveolar epithelial cells as a key initiator driving neutrophil recruitment during sepsis. ELISA quantification confirmed that pharmacological inhibition of RIPK1, RIPK3 or MLKL significantly reduced CXCL1 secretion in LPS‐stimulated alveolar epithelial cells (Figure S3G, Supporting Information), suggesting that RIPK1‐mediated necroptosis is involved in the extracellular release of CXCL1, further solidifying CXCL1 as a key downstream effector of RIPK1 signaling in alveolar epithelial cells.

CXCL1 (C‐X‐C motif chemokine ligand 1), a CXCR2‐binding chemokine pivotal for neutrophil chemotaxis,^[^ ^10^, ^45^ ^]^ is hyperactivated during dysregulated inflammation, driving pathogenic neutrophil influx that exacerbates tissue damage.^[^ ^7^ ^]^ While alveolar macrophages and endothelial cells contribute to chemokine production,^[^ ^5^, ^8^ ^]^ our integrated transcriptomic‐proteomic analysis identifies alveolar epithelial RIPK1 as a non‐redundant contributor of CXCL1. Specifically, siRNA‐mediated *Ripk1* knockdown reduced LPS‐induced CXCL1 expression in epithelial cells. This epithelial‐specific regulation mechanistically links RIPK1 to neutrophil‐mediated injury and positions RIPK1 as a critical orchestrator of CXCL1‐driven neutrophilic inflammation, highlighting its unique role in translating epithelial stress signals into alveolar injury during sepsis.

To establish a causal link between the RIPK1‐CXCL1 axis and neutrophil recruitment, we isolated Ly6G+ neutrophils from mouse bone marrow (>98% purity; Figure S4A,B, Supporting Information) and exposed them to conditioned media from LPS‐treated alveolar epithelial cells (Figure 4E). *Ripk1* knockdown in epithelial cells abrogated neutrophil migration in Transwell assays (Figure 4F), concomitant with reduction in CXCR2 receptor expression (Figure 4G), the primary chemokine receptor mediating neutrophil chemotaxis.^[^ ^46^, ^47^ ^]^ Furthermore, *Ripk1* silencing attenuated oxidative burst activity in neutrophils, evidenced by flow cytometry and western blot analysis of oxidative stress markers (Figure 4H; Figure S4C, Supporting Information). In vivo validation using ATII‐specific *Ripk1* knockout mice (*Ripk1* ^flox/flox^; *Sftpc*‐CreER/+) revealed a significantly reduced population of pulmonary CXCR2+ neutrophils compared to littermate controls post‐CLP (Figure 4I; Figure S4D,E, Supporting Information), closely mirroring in vitro findings of attenuated neutrophil migration (Figure 4F). These findings collectively demonstrate that RIPK1 in alveolar epithelial cells orchestrates neutrophil recruitment and activation through CXCL1‐CXCR2 signaling, providing a mechanistic basis for its role in amplifying inflammation during sepsis.

### 2.5 RIPK1 Orchestrates the JAK1‐STAT3 Signaling Axis to Facilitate CXCL1 Transcriptional Activation

**Figure 5:**
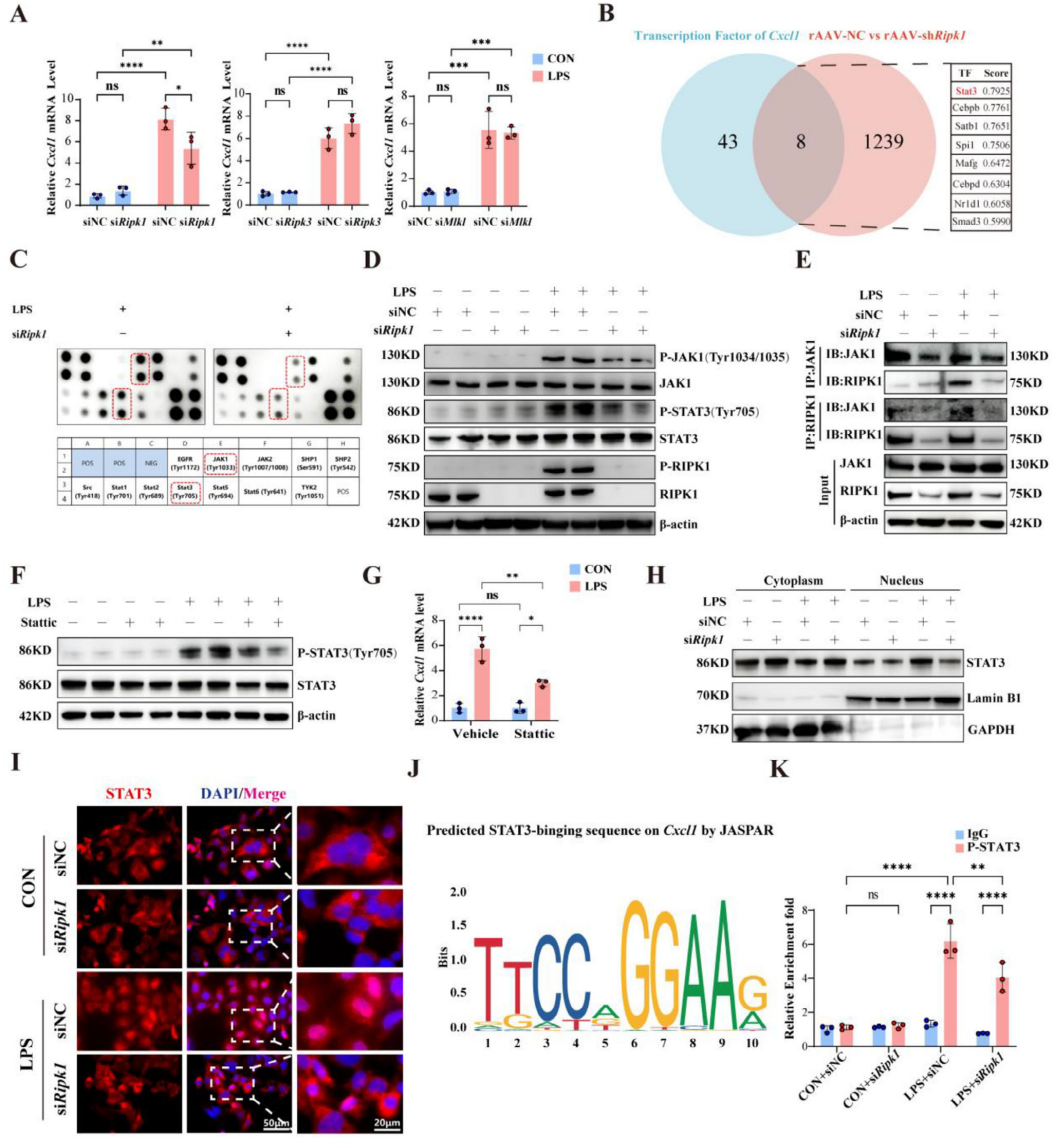
RIPK1 Orchestrates the JAK1‐STAT3 Signaling Axis to Facilitate CXCL1 Transcriptional Activation. A) Quantitative PCR analysis of *Cxcl1* mRNA levels in alveolar epithelial cells transfected with siNC, si*Ripk1*, si*Ripk3*, or si*Mlkl*, and stimulated with CON or LPS (n = 3). B) Identification of Transcription factor (TF) of CXCL1 with integrative analysis of transcriptomic sequencing and database within the CistromeDB toolkit. C) Protein array analysis of phosphorylated signaling proteins in alveolar epithelial cells treated with LPS and siRipk1. Red boxes highlight key phosphorylated proteins involved in the JAK/STAT pathway. D) Western blot analysis of phosphorylated JAK1 (P‐JAK1 Tyr1034/1035), total JAK1, phosphorylated STAT3 (P‐STAT3 Tyr705), total STAT3, phosphorylated RIPK1 (P‐RIPK1 S166), and total RIPK1 in alveolar epithelial cells transfected with siNC or si*Ripk1* and treated with or without LPS. β‐actin served as a loading control. E) Co‐immunoprecipitation analysis of interactions between RIPK1 and JAK1 in alveolar epithelial cells with or without si*Ripk1* and LPS stimulation. F) Western blot analysis of P‐STAT3(Tyr705) and total STAT3 in alveolar epithelial cells pre‐treated with the STAT3 inhibitor Stattic and stimulated with LPS. β‐actin served as a loading control. G) Quantitative PCR analysis of *Cxcl1* mRNA levels in alveolar epithelial cells treated with Stattic under LPS stimulation (n = 3). H) Western blot analysis of cytoplasmic and nuclear fractions of STAT3 in alveolar epithelial cells transfected with siNC or si*Ripk1* and treated with LPS. Lamin B1 and GAPDH served as nuclear and cytoplasmic loading controls, respectively. I) Immunofluorescence staining of STAT3 (red) and nuclei (DAPI, blue) in alveolar epithelial cells transfected with siNC or si*Ripk1* under CON or LPS conditions. Scale bar: 50 and 20µm. J) JASPAR‐predicted STAT3 binding motif on the *Cxcl1* promoter sequence, indicating potential transcriptional regulation sites. K) CUT&Tag‐qPCR analysis of P‐STAT3 enrichment at the *Cxcl1* promoter region in alveolar epithelial cells under different conditions. Data are presented as fold enrichment relative to IgG control (n = 3). Data are presented as mean ± SD. Multiple comparisons were analyzed using two‐way ANOVA (A, G, K). ****P < 0.0001;***P < 0.001; **P < 0.01; *P < 0.05; NS, no significant difference. SD, standard deviation; ANOVA, analysis of variance.

Consistent with transcriptomic profiling (Figure 4A), genetic knockdown of *Ripk1* attenuated LPS‐induced *Cxcl1* mRNA upregulation (Figure 5A). In contrast, *Ripk3* or *Mlkl* silencing did not result in significant effects (**Figure** **5**A), indicating the presence of alternative mechanisms or pathways by which RIPK1 governs CXCL1 production. The knockdown efficiencies of RIPK1, RIPK3, and MLKL were confirmed by western blot analysis (Figure S2I; Figure S5A,B, Supporting Information).

Since RIPK1 is not a transcription factor, we hypothesized that it regulates *Cxcl1* expression through intermediary signaling molecules that modulate transcriptional activity. To identify the transcription factors mediating this regulation, we performed an integrated bioinformatics analysis combining our *Ripk1* knockdown transcriptome data with the Toolkit for CistromeDB database. This approach revealed STAT3 as the most promising candidate transcription factor responsible for RIPK1‐dependent CXCL1 transcriptional regulation (Figure 5B). Guided by these predictions, we focus on the JAK‐STAT signaling pathway, where transcriptomic analysis showed significant enrichment and implicated *Jak1* and *Stat3* as potential RIPK1 targets (Figure S5C, Supporting Information). The results from the JAK/STAT pathway phosphorylation array and western blot further demonstrate that RIPK1 regulates the activation of the JAK1‐STAT3 pathway induced by LPS stimulation (Figure 5C,D). The STAT protein family, including STAT3, plays a crucial role in various biological processes such as cell proliferation, survival, differentiation, and angiogenesis.^[^ ^48^ ^]^ STAT3 is activated in response to various cytokines and growth factors predominantly via phosphorylation. Phosphorylated STAT3 translocates from cytoplasm to the nucleus, where it binds promoter regions to initiate target gene transcription.^[^ ^49^ ^]^ It is worth noting that the activation of STAT3 in the transcriptional regulation of *Cxcl1* has been implicated in the mechanisms of tumor diseases and immune disorders,^[^ ^23^, ^24^ ^]^ supporting our hypothesis that RIPK1 promotes *Cxcl1* expression via JAK1‐STAT3 signaling in AECs in response to inflammatory stimulation.

To elucidate RIPK1's role in JAK1‐STAT3 signaling, we aimed to examine the association between RIPK1 and JAK1. Based on the molecular docking analysis, the interaction between RIPK1 and JAK1 reveals a stable binding mode, with key amino acid residues forming multiple hydrogen bonds and hydrophobic interactions at the interface (Figure S5D, Supporting Information), indicating a strong and biologically relevant interaction. To investigate the specificity of RIPK1 interactions with JAK family members, we first performed co‐immunoprecipitation (Co‐IP) experiments to screen RIPK1 binding to all JAK family members under LPS stimulation. Our results showed that RIPK1 selectively bound to JAK1, with no significant interaction observed with other JAK family members such as JAK2, JAK3, or TYK2 (Figure S5E, Supporting Information). To further validate JAK1's critical role in RIPK1 downstream signaling, we knocked down JAK1 expression in AECs using siRNA (Figure S5F, Supporting Information). JAK1 knockdown significantly reduced LPS‐induced STAT3 phosphorylation and the *Cxcl1* mRNA expression(Figure S5G,H, Supporting Information), with no compensatory increase in JAK2 phosphorylation. These findings collectively confirm that JAK1 is essential and specific to RIPK1‐mediated signaling, driving STAT3 activation and *Cxcl1* expression exclusively through the JAK1 pathway. To establish the functional relevance of STAT3 activation, we inhibited STAT3 with Stattic,^[^ ^50^ ^]^ which abolished LPS‐induced STAT3 phosphorylation and *Cxcl1* mRNA upregulation (Figure 5F,G, Supporting Information). Consistent with these findings, LPS triggered STAT3 nuclear translocation, a process attenuated by *Ripk1* knockdown (Figure 5H,I). Using JASPAR, we identified a putative STAT3 binding site in the *Cxcl1* promoter and designed primers for targeted analysis (Figure 5J; Figure S5D, Supporting Information). Cut‐tag (Cleavage Under Targets and Tagmentation) in conjunction with qPCR revealed LPS‐induced P‐STAT3 occupancy at the *Cxcl1* promoter, which was suppressed by *Ripk1* silencing (Figure 5K; Figure S5I, Supporting Information). Our dual‐luciferase reporter assays in MLE‐12 cells directly demonstrate the functional significance of the predicted STAT3 binding site within the *Cxcl1* promoter. Co‐transfection with a STAT3 overexpression plasmid and the wild‐type *Cxcl1* promoter reporter significantly increased luciferase activity compared to controls, demonstrating STAT3‐driven *Cxcl1* transcription. Conversely, mutating the predicted STAT3 binding site within the *Cxcl1* promoter abrogated this enhancing effect (Figure S5J, Supporting Information). These findings collectively establish that the integrity of this identified STAT3 binding site is essential for STAT3‐mediated transcriptional activation of *Cxcl1*.

To further ascertain the specificity of the JAK1‐STAT3 pathway in the context of RIPK1‐mediated inflammation, we also evaluated the activation of other well‐established inflammatory signaling pathways. Western blot analysis of key components of the NF‐κB pathway (P‐NF‐κB p65) and major MAPK pathways (P‐ERK1/2, P‐p38 MAPK, P‐JNK) in LPS‐stimulated alveolar epithelial cells revealed no significant changes in their phosphorylation or activation status upon RIPK1 knockdown (Figure S5K, Supporting Information). These results highlight the JAK1‐STAT3 axis as the primary pathway regulated by RIPK1 in our experimental model.

### 2.6 A Novel RIPK1 Inhibitor Mitigates Sepsis‐Induced Lung Injury

Despite advancements in RIPK1 inhibitor (RIPK1i) development, clinical translation remains challenging due to limited selectivity, off‐target effects, suboptimal potency—especially against murine RIPK1—and poor pharmacokinetics, including low oral bioavailability and short half‐life.^[^ ^12^, ^27^, ^51^ ^]^ To address these limitations, we utilized an innovative RIPK1 inhibitor, compound 62(C62), which effectively addresses several limitations of current inhibitors. With subnanomolar EC50 values and an IC50 of 3.5 nmolL^−1^ in vitro kinase assays, it demonstrates improved selectivity and potency against both human and murine RIPK1. Additionally, it exhibits favorable pharmacokinetic properties, including excellent oral bioavailability and an extended half‐life. Both in vitro and in vivo studies confirm its ability to potently suppress RIPK1‐mediated inflammatory pathways.^[^ ^29^ ^]^ To assess the safety profile of Compound 62, mice were orally administered 30 mgkg^−1^ daily for seven consecutive days. Serum biochemical analysis revealed no significant alterations in liver or kidney function markers (Figure S6A, Supporting Information). Histopathological evaluation of lung, liver, and kidney tissues via H&E staining further supported the compound's favorable safety profile, with no observable pathological changes in any of the examined organs(Figure S6B, Supporting Information).

**Figure 6:**
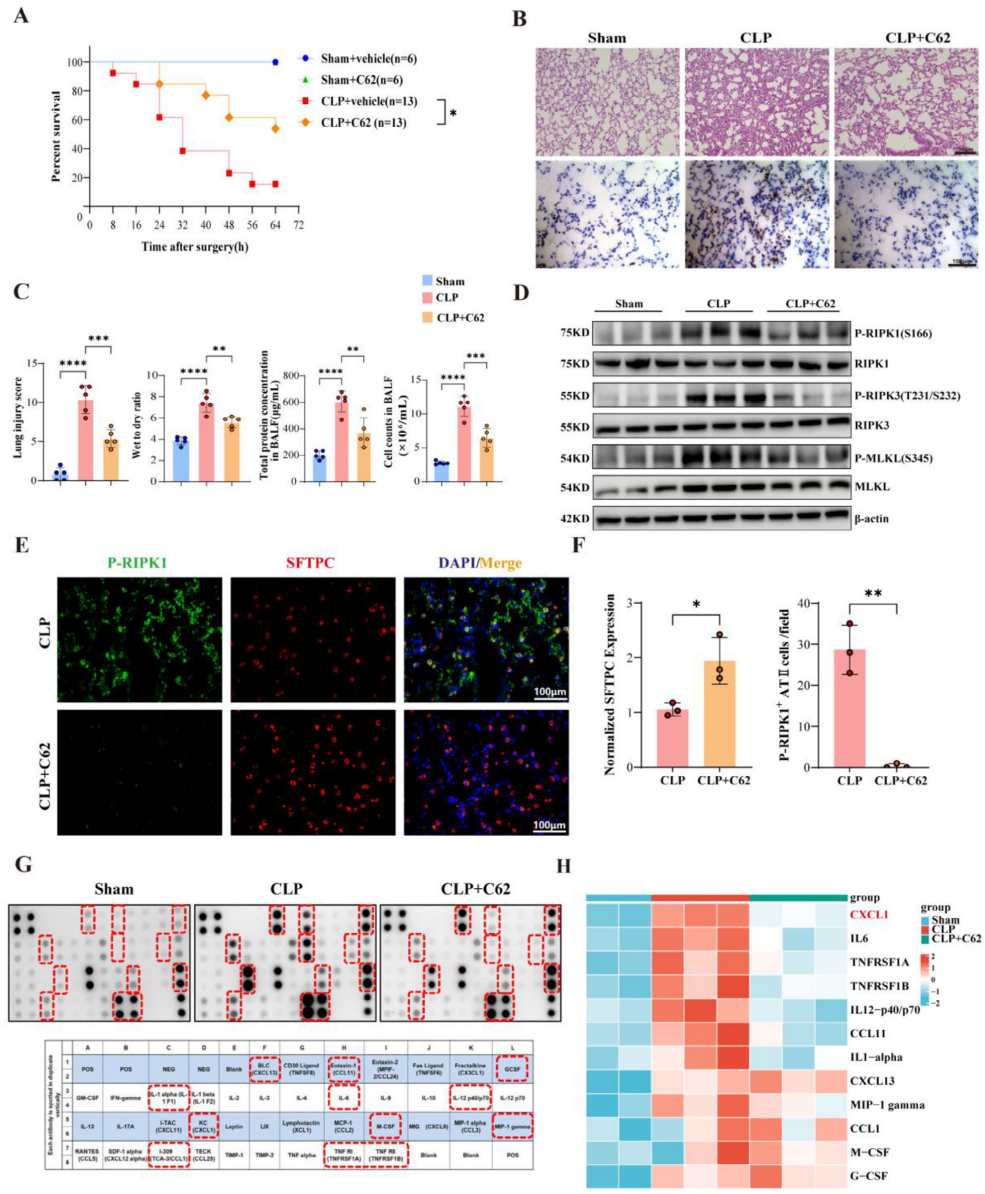
A Novel RIPK1 Inhibitor Mitigates Sepsis‐Induced Lung Injury. A) Kaplan‐Meier survival curves showing the percentage survival of mice subjected to sham or CLP surgery and treated with vehicle or RIPK1 inhibitor (62) over 72 h (Sham groups, n = 6; CLP groups, n = 13). B) Representative H&E‐stained lung tissue sections (top panel, scale bar: 50µm) and immunohistochemical staining for neutrophils (bottom panel, scale bar: 100µm) in sham, CLP, and CLP+C62 groups. C) Quantification of lung injury scores, wet‐to‐dry weight ratios, total protein concentration in bronchoalveolar lavage fluid (BALF), and cell counts in BALF across different treatment groups (n = 5). D) Western blot analysis of lung tissue lysates showing the expression of phosphorylated RIPK1 (P‐RIPK1 S166), total RIPK1, phosphorylated RIPK3 (P‐RIPK3 T231/S232), total RIPK3, phosphorylated MLKL (P‐MLKL S345), and total MLKL. β‐actin served as a loading control. E) Representative immunofluorescence images of P‐RIPK1 (green), SFTPC (red), and nuclei (DAPI, blue) in lung tissues from CLP and CLP+C62 groups. Scale bar: 100 µm. F) Quantification of normalized SFTPC fluorescence intensity and the number of P‐RIPK1+ SFTPC+ cells per field in CLP and CLP+C62 groups (n = 3). G) Protein array analysis of inflammatory mediators in lung tissues from sham, CLP, and CLP+C62 groups. Red dashed boxes highlight differentially regulated cytokines and chemokines. (H) Heatmap representation of key inflammatory cytokines and chemokines in serum from sham, CLP, and CLP+C62 groups, highlighting CXCL1 as a prominent factor. Data are presented as mean ± SD. Survival comparisons were analyzed using the log‐rank test (A). Multiple comparisons were analyzed using one‐way ANOVA (C). Two‐group comparisons were analyzed using Student's *t*‐test (F). ****P < 0.0001;***P < 0.001; **P < 0.01; *P < 0.05; NS, no significant difference. SD, standard deviation; ANOVA, analysis of variance.

Compound 62 demonstrates significant therapeutic potential in treating sepsis‐induced lung injury, as evidenced by improved survival rates in both cecal ligation and puncture (CLP) and lipopolysaccharide (LPS)‐induced sepsis models. In prophylactic studies, oral administration of Compound 62 just 15 min before either CLP or LPS challenge significantly enhanced survival (**Figure** **6**A; Figure S7A, Supporting Information). Building on these promising results, we further investigated its therapeutic efficacy by administering Compound 62 orally 3 h after CLP surgery (Figure S7B, Supporting Information). This post‐treatment regimen also resulted in a significant improvement in survival, reinforcing the prophylactic findings and highlighting Compound 62′s potential as an effctive intervention for sepsis. More importantly, sepsis‐induced lung injury was also effectively alleviated. This improvement was evidenced by reduced pathological damage in lung tissue and decreased neutrophil infiltration (Figure 6B), along with lower total protein concentration and cell counts in BALF, as well as a reduced lung wet‐to‐dry weight ratio (Figure 6C). Additionally, apoptosis of lung cells was significantly attenuated, further supporting the protective effect of the treatment (Figure S7C,D, Supporting Information).

Further investigations were conducted to precisely characterize the influence of Compound 62 on neutrophil behavior. Histological assessments, specifically utilizing MPO immunofluorescence staining, provided compelling visual evidence of significantly diminished neutrophil activation in lung tissues following Compound 62 administration in vivo (Figure S8A, Supporting Information), consistent with Ly6G+ immunohistochemistry findings (Figure 6B). Moreover, in vitro studies employing conditioned media derived from alveolar epithelial cells pre‐treated with Compound 62 revealed a notable impairment in both neutrophil chemotaxis and activation (Figure S8D, Supporting Information). Collectively, these multifaceted results unequivocally establish that Compound 62 exerts a direct regulatory effect on neutrophil activation and migratory capabilities, thereby contributing to its established therapeutic efficacy in alleviating sepsis‐induced lung injury.

Mechanistically, compound 62 inhibited RIPK1 activation and downstream inflammatory signaling in septic lungs, while the loss of type II alveolar epithelial cells was markedly alleviated (Figure 6D–F, Supporting Information). Semi‐quantitative cytokine array analysis of mouse serum collected 24 hours post‐CLP revealed that compound 62 significantly suppressed the elevated expression of pro‐inflammatory cytokines and chemokines. Notably, CXCL1 was identified as a key inhibited factor (Figure 6G,H), further corroborating our previous findings. In summary, our results indicate that compound 62 mitigates sepsis‐induced lung injury by targeting RIPK1‐mediated inflammatory cascades.

## 3 Discussion

Sepsis‐induced lung injury, characterized by dysregulated inflammation and alveolar epithelial damage, poses a significant clinical challenge.^[^ ^2^, ^3^ ^]^ This study reveals a novel mechanism by which receptor‐interacting serine/threonine‐protein kinase 1 (RIPK1) drives neutrophil‐mediated lung injury through the JAK1‐STAT3‐CXCL1 signaling axis in alveolar epithelial cells (AECs), highlighting RIPK1's functionality beyond necroptosis and redefining alveolar structural cells as active contributors to inflammatory amplification.

RIPK1, traditionally associated with necroptosis via RIPK3‐MLKL activation,^[^ ^11^, ^13^ ^]^ possesses kinase activity that regulates CXCL1 production in AECs independently of necroptosis. Our findings demonstrate that RIPK1 interacts with JAK1, facilitating STAT3 phosphorylation and nuclear translocation. Activated STAT3 directly binds the *Cxcl1* promoter, driving its transcription and subsequent secretion of CXCL1, a potent neutrophil chemoattractant.^[^ ^10^, ^52^, ^53^, ^54^ ^]^ This pathway operates independently of necroptosis, as demonstrated by the absence of CXCL1 regulation in RIPK3‐ or MLKL‐deficient cells. CXCL1 plays a central role in neutrophil mobilization and recruitment during bacterial pneumonia‐induced sepsis.^[^ ^9^ ^]^ Our previous studies have demonstrated a significant upregulation of CXCL1 expression in the hippocampus of septic mice,^[^ ^55^ ^]^ suggesting its critical involvement in the signaling network that drives the sepsis‐associated cytokine storm. Genetic ablation (*Ripk1* ^CKO^ mice) or pharmacological inhibition (compound 62) of RIPK1 attenuated CXCL1 expression, neutrophil infiltration, and alveolar damage, underscoring RIPK1's critical role in this axis. Our findings present a novel perspective, contrasting with prior in vivo and clinical studies that primarily focused on macrophage‐ or endothelial‐derived active ingredients as primary drivers of neutrophilic inflammation in lung diseases.^[^ ^17^, ^18^, ^56^, ^57^, ^58^, ^59^ ^]^ By identifying AECs as one of the important sources of CXCL1, our work challenges the paradigm of immune cell dominance, highlighting the active and crucial involvement of structural cells in inflammatory signaling. These findings are not intended to negate the contributions of other cell types but rather to emphasize that within the specific pathophysiological context of septic lung injury, AECs represent a major and previously underestimated source of CXCL1.

The discovery of RIPK1‐JAK1 interaction introduces kinase cross‐talk as a novel regulatory mechanism for JAK‐STAT activation, which has historically been attributed to cytokine receptor signaling. This finding aligns with emerging evidence of RIPK1's non‐necroptotic roles but expands its scope to include transcriptional regulation of chemokines. Furthermore, the development of compound 62, a selective RIPK1 inhibitor with improved pharmacokinetics,^[^ ^29^ ^]^ addresses a critical gap in sepsis therapeutics. Unlike earlier inhibitors such as Necrostatin‐1, which exhibited off‐target effects and metabolic instability,^[^ ^27^, ^28^, ^60^ ^]^ compound 62 selectively suppresses RIPK1‐mediated inflammation without broadly disrupting other cell death pathways. This precision‐targeted approach effectively mitigates lung injury while preserving epithelial integrity.

Despite these advances, our study has limitations. First, the reliance on murine models (CLP and LPS) restricts direct translation to human sepsis, which exhibits greater etiological and immunological heterogeneity.^[^ ^61^, ^62^, ^63^, ^64^ ^]^ While plasma P‐RIPK1 levels correlated with disease severity in sepsis patients, direct evidence of the JAK1‐STAT3‐CXCL1 axis in human lungs remains to be established. Second, the focus on AECs overlooks potential contributions from other RIPK1‐expressing cells, such as macrophages or endothelial cells, which may modulate inflammation through parallel pathways. Third, suppression of RIPK1 could impair NF‐κB‐mediated survival signals,^[^ ^13^, ^65^ ^]^ necessitating careful evaluation in preclinical models. Although we have validated the safety profile of short‐term alveolar epithelial cell‐specific RIPK1 knockout, the long‐term implications of sustained RIPK1 inhibition, particularly its regulatory effects on epithelial regeneration and pulmonary immune homeostasis,^[^ ^22^, ^66^, ^67^ ^]^ require systematic evaluation using well‐established chronic infection models.

Beyond the pivotal role of CXCL1 delineated in our RIPK1‐mediated inflammatory cascade, our data revealing the concomitant upregulation of CXCL5 and CCL20 warrants consideration of their combinatorial contributions. Critically, CXCL5, like CXCL1, is a potent CXCR2 agonist.^[^ ^68^, ^69^ ^]^ Their co‐expression likely engenders synergistic amplification of neutrophil chemotaxis and activation, potentially exceeding the effect elicited by either chemokine alone. This synergy, stemming from shared receptor engagement and possible cooperative binding or signaling dynamics, provides a plausible mechanism for the robust and sustained neutrophilic infiltration observed in our model, culminating in tissue injury. While CCL20 operates primarily via CCR6, recruiting distinct leukocyte subsets (e.g., Th17 cells, dendritic cells),^[^ ^68^, ^70^ ^]^ its co‐upregulation suggests RIPK1 orchestrates a broader inflammatory milieu. CCL20 may contribute to amplifying or diversifying the immune response, potentially interacting with CXCL1/CXCL5‐driven pathways to exacerbate inflammation or influence its resolution. Elucidating these intricate interactions will not only refine our understanding of RIPK1‐driven immunopathology but also inform the development of more targeted therapeutic strategies aimed at modulating specific chemokine axes within the inflammatory network.

Future research should prioritize translational validation of these findings. Testing compound 62 in humanized models, such as lung organoids or immunocompetent mice engrafted with human cells, would bridge the gap between murine data and human physiology. Combinatorial approaches—pairing RIPK1 inhibitors with CXCR2 antagonists or JAK1 blockers—may enhance therapeutic efficacy while reducing off‐target effects. Beyond bacterial sepsis, exploring RIPK1's role in viral or fungal models could elucidate its universality across sepsis etiologies. Finally, investigating RIPK1's contribution to chronic sequelae, such as post‐sepsis fibrosis and immune disorder, may uncover broader implications for critical care outcomes. Existing research indicates that RIPK1 plays a critical role in maintaining T cell homeostasis by preventing TNFR1‐induced apoptosis and RIPK3/caspase‐8‐mediated senescence,^[^ ^71^, ^72^ ^]^ thereby safeguarding against immune dysregulation and inflammation. Consequently, its involvement in the immunosuppressive pathology of late‐stage sepsis warrants further exploration.

In conclusion, this study repositions RIPK1 as a pleiotropic regulator of sepsis‐induced lung injury, bridging necroptosis, and chemokine‐driven inflammation. By elucidating the RIPK1‐JAK1‐STAT3‐CXCL1 axis in alveolar epithelial cells, we challenge traditional narratives of immune cell‐centric inflammation and provide a mechanistic foundation for precision therapies. While compound 62 exemplifies the promise of kinase‐specific targeting, addressing translational gaps and optimizing combinatorial strategies will be essential to advance sepsis management. These findings not only refine RIPK1's biological narrative but also underscore the therapeutic potential of redefining structural cells as active players in inflammatory pathology.

## 4 Experimental Section

### Study Design

The objective of this study was to elucidate the role and underlying molecular mechanisms of RIPK1 in mediating CXCL1 production and subsequent neutrophil recruitment during sepsis‐induced acute lung injury (ALI). Integrating clinical data from sepsis patients with murine models of cecal ligation and puncture (CLP) and LPS‐induced endotoxemia, RIPK1 activation dynamics and its mechanistic contributions were investigated RIPK1 activation dynamics and its mechanistic contributions. Human plasma Phospho‐RIPK1 (Figure S1, Supporting Information) levels were correlated with disease severity and respiratory dysfunction. Mechanistic studies employed alveolar epithelial cell‐specific *Ripk1* knockout mice (*Sftpc*‐CreER/+; *Ripk1* ^flox/flox^), systemic kinase‐dead *Ripk1* ^D138N/D138N^ mutants, and pharmacological inhibition using the selective RIPK1 inhibitor Compound 62. In vitro models utilized LPS‐stimulated MLE‐12 alveolar epithelial cells and bone marrow‐derived neutrophils to dissect RIPK1‐JAK1‐STAT3‐CXCL1 signaling via transcriptomic/proteomic profiling, co‐immunoprecipitation, chromatin occupancy assays (CUT&Tag‐qPCR), and functional migration assays. Therapeutic efficacy was assessed through survival analysis, histopathology, and inflammatory mediator quantification. The experimental parameters for in vitro studies were established based on previously published literature and preliminary testing. Sample sizes for in vitro assays followed standards commonly used in the field by other research groups. Throughout all experiments involving mice, cells, histology, or other analyses, investigators were blinded to sample identities prior to image acquisition and data quantification. No data were excluded from any study. Detailed information regarding sample sizes and the number of experimental replicates for each assay is provided in the corresponding Figure legends.

### Clinical Specimens

This study enrolled 11 patients diagnosed with acute‐phase sepsis requiring intensive care unit (ICU) admission, following ethical approval from the Institutional Review Board of Zhongshan Hospital, Fudan University (Ethical Approval Number: B2021‐182R) and written informed consent from participants or legal representatives. Inclusion criteria, aligned with Sepsis‐3.0 guidelines,^[^ ^2^, ^3^ ^]^ comprised: 1) age 18–80 years; 2) Sequential Organ Failure Assessment (SOFA) score ≥ 2 (calculated using 24‐h worst parameters); 3) meeting ≥ 2 systemic inflammatory response syndrome (SIRS) criteria (temperature > 38 °C/< 36 °C, heart rate > 90 beats/min, respiratory rate > 20 breaths/min or PaCO_2_ < 32 mmHg, leukocyte count > 10×10⁹/L/< 4×10⁹/L, or immature granulocytes > 10%); and 4) requirement for ≥ 4 days of intravenous antibiotics. Exclusion criteria included malignancies, autoimmune diseases, recent transfusion/active bleeding, acute cardiocerebrovascular events, immunosuppressive therapy, pregnancy/lactation, survival expectancy < 48 h, concurrent clinical trial participation, or refusal to enroll. Baseline demographic (sex, age, BMI), clinical (SOFA, APACHE II scores, vital signs), and laboratory parameters (CRP, leukocyte/lymphocyte counts) were systematically documented (Table S1, Supporting Information). Peripheral blood processing involved collecting 5 mL EDTA‐anticoagulated blood within 24 h of diagnosis. After 20‐min room‐temperature stabilization, samples were centrifuged at 1500 ×g for 20 min to isolate plasma, which was aliquoted into cryovials, flash‐frozen in liquid nitrogen, and stored at −80 °C for analysis.

### Mice

Male C57BL/6J mice (8–10 weeks old, 20–25g) were obtained from Shanghai Laboratory Animal Research Center. *Ripk1* ^D138N/D138N^ mice were generated and authorized for use by Prof. Junying Yuan's research group at the Center for Interdisciplinary Research in Biology and Chemistry, Shanghai Institute of Organic Chemistry, Chinese Academy of Sciences(CAS). *Sftpc*‐CreER/+ mice were generously provided by Prof. Bin Zhou from the Institute of Biochemistry and Cell Biology, Shanghai Institute for Biological Sciences, CAS. *Ripk1* ^flox/flox^ mice were generously provided by Prof. Haibing Zhang from the Shanghai Institute of Nutrition and Health, CAS. All mice were housed under specific pathogen‐free conditions (22–24 °C, 50–60% humidity, 12‐h light/dark cycle) with ad libitum access to food and water. All animal experimental procedures conducted in this study were reviewed and approved by the Animal Ethics Committee of Zhongshan Hospital, Fudan University (Ethical Approval Number: 2020–119).

### Sepsis Models

Sepsis was induced using two well‐established murine models: cecal ligation and puncture (CLP) and lipopolysaccharide (LPS) challenge.^[^ ^64^ ^]^

### Cecal Ligation and Puncture (CLP) Model

Briefly, mice were anesthetized via intraperitoneal injection of pentobarbital sodium (50 mgkg^−1^). Following a midline laparotomy, the cecum was exteriorized and ligated at two‐thirds of its length proximal to the ileocecal valve, then punctured once using an 18‐gauge needle to extrude a minimal fecal content (≈1 mm^3^). Sham‐operated mice underwent an identical laparotomy without cecal ligation or puncture. Postoperatively, mice received subcutaneous saline resuscitation (1 mL) and analgesia (buprenorphine, 0.1 mgkg^−1^). Survival was monitored every 8 h for 72 h, and moribund mice (defined by labored breathing, unresponsiveness, or inability to ambulate) were euthanized via cervical dislocation under anesthesia. Lung, liver, and kidney tissues were harvested 24 h post‐CLP for downstream analyses.

### LPS‐Induced Sepsis Model

For endotoxemia‐induced sepsis, mice were administered an intraperitoneal injection of LPS (10 mgkg^−1^, Escherichia coli O55:B5, Sigma–Aldrich, L2880) dissolved in sterile phosphate‐buffered saline (PBS). Mice were monitored once daily for clinical signs of sepsis and survival over a 7‐day period.

### Assessment of Sepsis Severity Using Modified Murine Sepsis Score (MSS)

To monitor disease progression and establish surrogate endpoints for mortality, a modified Murine Sepsis Score (MSS)^[^ ^31^ ^]^ adapted from Shrum et al. (2014) was employed.^[^ ^30^ ^]^ This scoring system evaluated six clinical parameters: appearance (coat condition), level of consciousness, spontaneous activity, response to external stimuli (auditory/tactile), eye aspect (closure/secretions), and respiratory quality (labored breathing/gasping). Each parameter was graded on a standardized four‐point scale (0 = normal, 1 = mild impairment, 2 = moderate impairment, 3 = severe impairment). The original respiratory rate component was excluded due to impractical visual quantification in the CLP model over a 24‐h period. Assessments were performed every 4 h post‐operatively by two independent blinded observers, with final scores representing the mean value across all components. Mice were briefly anesthetized with isoflurane (<2 min) during scoring to ensure consistency.

### Intrapulmonary Delivery of Recombinant Adeno‐Associated Virus

Recombinant adeno‐associated virus (rAAV) was delivered to murine lungs via intratracheal instillation using rAAV6‐U6‐shRNA(*Ripk1*)‐CMV‐EGFP‐WPRE or rAAV6‐U6‐shRNA (NC)‐CMV‐EGFP‐WPRE constructs. Eight‐ to ten‐week‐old male C57BL/6J mice (body weight 20–25 g) were anesthetized in an isoflurane induction chamber (4–5% isoflurane + 95% O_2_). Upon loss of voluntary movement, mice were transferred to a surgical platform and maintained under 1–2% isoflurane via a nose cone to ensure stable respiration and analgesia. Mice were secured in a supine position on a 45°angled platform. A sterile cotton swab was used to gently extend the tongue for oropharyngeal exposure. Transillumination of the oral cavity facilitated visualization of the tracheal opening. A 22‐gauge catheter was carefully advanced into the trachea (insertion depth: 0.5 cm), with correct positioning verified by observing respiratory airflow within the catheter or using a stethoscope. Viral suspensions (3×10^12^ vgmL^−1^ in 50 µL sterile PBS) were slowly instilled via a microsyringe at a controlled rate of 10 µLs^−1^ to prevent esophageal reflux or airway obstruction. Post‐instillation, mice were maintained in a tilted position for 30 s to optimize viral distribution. Catheters were gently withdrawn after confirming spontaneous respiratory recovery, and mice were recovered on a warming pad for ≥2 h with continuous respiratory monitoring.

### Hematoxylin and Eosin (HE) Staining

Lungs, Livers, and Kidneys were collected at the indicated time points, perfused with phosphate‐buffered saline (PBS) to remove blood residues, and fixed in 4% paraformaldehyde (PFA) at 4 °C for 24 h. Following fixation, tissues were dehydrated, embedded in paraffin, and sectioned at a thickness of 5 µm using a rotary microtome (Leica RM2235). Tissue sections were deparaffinized in xylene and rehydrated through a descending ethanol series before staining with hematoxylin for 3 min. After rinsing in running tap water, sections were differentiated in 1% acid alcohol for 10 s and subsequently counterstained with eosin for 30 s. Stained sections were then dehydrated through an ascending ethanol series, cleared in xylene, and mounted using a neutral resin. Histopathological analysis of lung tissues was meticulously conducted using a bright‐field microscope (Keyence BZ‐X series). To objectively quantify the severity of acute lung injury (ALI), a well‐established semi‐quantitative histological scoring system was employed, as previously validated.^[^ ^2^, ^3^ ^]^ This system evaluated four key pathological features: alveolar edema (accumulation of proteinaceous fluid within alveolar spaces), hemorrhage (extent of red blood cell extravasation into alveoli and interstitium), leukocyte infiltration (density of inflammatory cells, predominantly neutrophils and lymphocytes, in alveolar and interstitial compartments), and alveolar wall thickening (widening of alveolar septa reflecting interstitial edema, inflammation, or early fibrosis). Two experienced pathologists, blinded to experimental groups, independently graded each parameter on a 0–3 scale: 0 (normal/absent pathology), 1 (mild/focal changes), 2 (moderate changes in substantial areas), or 3 (severe/widespread damage). Individual parameter scores were summed to yield a total lung injury score per sample (range: 0–12). Scoring discrepancies were resolved through consensus review. Final scores per experimental group were used for statistical analysis, with higher values indicating more severe tissue injury.

### Immunofluorescence (IF) Staining

For immunofluorescence analysis, lung tissues were embedded in optimal cutting temperature (OCT) compound and rapidly frozen in liquid nitrogen. Cryosections of 6–8 µm thickness were prepared using a cryostat (Leica CM1950) and mounted on poly‐L‐lysine‐coated slides. Sections were fixed in 4% paraformaldehyde (PFA) for 15 min at room temperature, followed by permeabilization with 0.2% Triton X‐100 in PBS for 10 min. To prevent non‐specific binding, sections were blocked using 10% normal goat serum in PBS for 1 h at room temperature. Primary antibodies, including P‐RIPK1 (Figure S1, Supporting Information) (Arigo Biolaboratories, Cat# ARG66476), SFTPC (Abcam, Cat# ab211326), AQP5 (Abcam, Cat# ab305303), CD45 (Abcam, Cat# ab317446), CD31 (Abcam, Cat# ab182981), F4/80 (Abcam, Cat# ab6640), CXCL1 (Thermo Fisher, Cat# PA5‐86508), Ly6G (BioLegend, Cat# 127 601), and CXCR2 (R&D Systems, Cat# MAB2164), were diluted in blocking solution and incubated overnight at 4 °C. The following day, sections were washed three times in PBS and incubated with the relevant Fluor‐conjugated secondary antibodies at room temperature for 1 h in the dark. Nuclei were counterstained with DAPI (SouthernBiotech, Cat# 0100–20) for 10 min, and slides were mounted with antifade mounting medium (Vector Laboratories, Cat# H‐1000). Imaging was performed using an all‐in‐one fluorescence microscope (Keyence BZ‐X series) with identical settings applied across all groups. Image analysis, including colocalization and fluorescence intensity measurements, was conducted using ImageJ (NIH).

### Immunohistochemistry (IHC)

For immunohistochemical analysis, paraffin‐embedded lung sections (4 µm) were deparaffinized in xylene and rehydrated through a descending ethanol series. Heat‐induced antigen retrieval was performed by immersing sections in sodium citrate buffer (10 mM, pH 6.0) and heating at 95 °C for 15 min. Endogenous peroxidase activity was quenched using 3% hydrogen peroxide for 10 min at room temperature. Sections were blocked with 5% bovine serum albumin (BSA) for 1 h before overnight incubation at 4 °C with primary antibodies targeting P‐RIPK1(Figure S1, Supporting Information) (Arigo Biolaboratories, Cat# ARG66476) and Ly6G (BioLegend, Cat# 127 601). After washing with PBS, sections were incubated with horseradish peroxidase (HRP)‐conjugated secondary antibodies for 1 h at room temperature. Immunoreactivity was visualized using a diaminobenzidine (DAB) substrate kit (Vector Laboratories, Cat# SK‐4100), followed by counterstaining with hematoxylin. After dehydration and mounting, stained sections were imaged using a bright‐field microscope (Keyence BZ‐X series).

### TUNEL Staining

Frozen lung sections (10 µm) were fixed in 4% paraformaldehyde for 20 min at room temperature, followed by PBS washing and permeabilization with 0.1% Triton X‐100 for 10 min. TUNEL staining was performed using the TUNEL Apoptosis Detection Kit (Beyotime, Cat# C1086) according to the manufacturer's protocol. Sections were incubated with the TUNEL reaction mixture at 37 °C for 1 h, washed with PBS, and counterstained with DAPI (SouthernBiotech, Cat# 0100–20). Images were captured using a fluorescence microscope (Keyence BZ‐X series).

### Lung Wet‐to‐Dry Ratio

The lung wet‐to‐dry (W/D) ratio was measured to assess pulmonary edema. Mice were euthanized, and the left lung lobes were carefully excised, rinsed with PBS to remove blood, and blotted dry with filter paper. The wet weight of the lungs was immediately recorded using an analytical balance. The samples were then placed in a drying oven at 60 °C for 72 h to ensure complete dehydration before measuring the dry weight. The W/D ratio was calculated by dividing the wet weight by the dry weight, providing an index of lung water content.

### Bronchoalveolar Lavage Fluid (BALF) Analysis

BALF was collected by instilling 1 mL of ice‐cold PBS into the trachea via a 22‐gauge catheter, followed by three gentle aspiration cycles. Total protein concentration was measured using a BCA assay (YEASEN, Cat# 20201ES76). Total cell counts were determined using an electronic cell counter (Thermo Fisher, Countess II FL, Cat# AMQAF1000).

### Cell Culture

The murine alveolar type II epithelial cell line MLE‐12 (ATCC® CRL‐2110, validated by STR profiling) was cultured in Ham's F‐12K medium (Gibco, Cat# 21 127 022) supplemented with 10% fetal bovine serum (FBS, Gibco, Cat# 26 140 079), 1% penicillin‐streptomycin (Gibco, Cat# 15 140 122) at 37 °C in a 5% CO_2_ humidified incubator. Bone marrow‐derived neutrophils were maintained in RPMI‐1640 medium (Gibco, Cat# 11 875 093) containing 10% FBS for ≤ 6 h to preserve viability.^[^ ^73^ ^]^ MLE‐12 cells were stimulated with ultrapure LPS (Escherichia coli O55:B5, Sigma–Aldrich, L6529) at 1 µgmL^−1^ for 24 h.

### siRNA‐Mediated Gene Knockdown

MLE‐12 cells were transfected with siRNAs targeting *Ripk1*, *Ripk3*, and *Mlkl* or a non‐targeting control siRNA (GenePharma, Shanghai, China) at a final siRNA concentration of 50 nM using Lipofectamine 3000 (Thermo Fisher, Cat# L3000008) according to the manufacturer's instructions. Knockdown efficiency was validated 48 h post‐transfection via Western blot, with HRP‐conjugated β‐actin (Proteintech, Cat# HRP‐60008) as a loading control.

### Cell Slide Immunofluorescence

MLE‐12 cells were seeded on sterile glass coverslips in a 24‐well plate and stimulated with 1 µgmL^−1^ LPS for 24 h. After treatment, cells were fixed with 4% PFA for 15 min, permeabilized with 0.5% Triton X‐100 for 15 min, and blocked with 5% BSA for 1 h at room temperature. Cells were incubated overnight at 4 °C with primary antibodies: P‐RIPK1 (Figure S1, Supporting Information) (Arigo Biolaboratories, Cat# ARG66476), STAT3 (Proteintech, Cat# 10253‐2‐AP), and CXCL1 (Thermo Fisher, Cat# PA5‐86508), followed by Alexa Fluor 488‐ or 555‐ conjugated secondary antibodies for 1 h at room temperature in the dark. Nuclei were counterstained with DAPI (SouthernBiotech, Cat# 0100–20) for 10 min, and coverslips were mounted with anti‐fade mounting medium. Fluorescence images were acquired using a fluorescence microscope (Keyence BZ‐X series).

### Cell Isolation and Sorting‐Isolation of Pulmonary Epithelial Cells

Lung tissues were harvested from 8‐week‐old male mice and immediately placed in ice‐cold phosphate‐buffered saline (PBS) to remove excess blood. The tissues were finely minced into ≈2–3 mm^3^ pieces and enzymatically digested using the Miltenyi Lung Tissue Dissociation Kit (Miltenyi Biotec, Cat# 130‐095‐927). The digestion reaction included Collagenase IV and DNase I and was performed at 37 °C for 30 min using the gentleMACS Dissociator (Miltenyi Biotec, Cat# 130‐093‐235) with the pre‐set program m_lung_01, following the manufacturer's protocol. After enzymatic digestion, the suspension was filtered through a 70‐µm cell strainer (Corning, Cat# 352 350) to remove undigested tissue fragments. The filtrate was then centrifuged at 300×g for 5 min at 4 °C to pellet the cells. The supernatant was discarded, and the cell pellet was resuspended in PBS containing 0.5% bovine serum albumin (BSA) to minimize cell aggregation. For pulmonary epithelial cell enrichment, single‐cell suspensions were incubated with CD326 (EpCAM) MicroBeads (Miltenyi Biotec, Cat# 130‐105‐958) for 15 min at 4 °C. The labeled cells were then passed through an LS Column (Miltenyi Biotec) placed in a magnetic field using the MACS Separator (Miltenyi Biotec), allowing the selective isolation of EpCAM‐positive epithelial cells. The purity of the sorted epithelial cells was validated by Western blot analysis, probing for EpCAM (epithelial cell marker), surfactant protein C (SFTPC) (alveolar type II cell marker), and aquaporin 5 (AQP5) (alveolar type I cell marker).

### Isolation of Bone Marrow‐Derived Neutrophils

Neutrophils were isolated from the bone marrow of 8‐week‐old male C57BL/6J mice. The femurs and tibias were carefully excised, and the bone marrow was flushed using pre‐chilled phosphate‐buffered saline (PBS) supplemented with 3% fetal bovine serum (FBS) to maintain cell viability. The marrow was flushed thoroughly to release the cells, ensuring a single‐cell suspension. The collected bone marrow cells were then passed through a 40‐µm cell strainer (Corning, Cat# 352 340) to remove any remaining clumps and debris, ensuring only single cells were retained for subsequent processing. To enrich the neutrophil fraction, the cells were subjected to density gradient centrifugation using Percoll, with two layers (52% and 64%) (Cytiva, Cat# 17‐0891‐01). The centrifugation was performed at 400 × g for 30 min at 4 °C to allow optimal separation of neutrophils from other cell types based on their density. Following centrifugation, the neutrophil‐enriched fraction was carefully collected from the interface of the Percoll layers. For further purification of neutrophils, magnetic‐activated cell sorting (MACS) was performed using Mouse Ly6G MicroBeads (Miltenyi Biotec, Cat# 130‐120‐337) according to the manufacturer's protocol. The MACS columns were prepared following the manufacturer's instructions, and cells were passed through the column to capture Ly6G‐positive neutrophils. After separation, the non‐bound cells were removed, and the neutrophils were eluted for further use. The purity of the isolated neutrophil population was assessed by flow cytometry, using specific markers such as Ly6G and CD11b to confirm the identity and purity of the neutrophils.

### Flow Cytometry

Single‐cell suspensions of lung tissue were prepared, and neutrophils were collected after experimental treatments. Cells were centrifuged at 400 ×g for 5 min at room temperature and resuspended in cell staining buffer (BioLegend, Cat# 420 201). To block non‐specific binding, cells were incubated with an anti‐CD16/32 antibody (BioLegend, Cat# 101 320) for 10 min at room temperature. Cell viability was assessed using Fixable Viability Stain 510 (BD Biosciences, Cat# 564 406), followed by a 30‐min incubation at room temperature in the dark. For surface staining, cells were incubated with the following fluorochrome‐conjugated antibodies:CD45 (PerCP‐Cy5.5‐conjugated, BioLegend, Cat# 103 132), CD11b (FITC‐conjugated, BioLegend, Cat# 101 206), Ly6G (PE/Cy7‐conjugated, BioLegend, Cat# 127 624), CXCR2 (PE‐conjugated, BioLegend, Cat# 304 012), Dihydrorhodamine 123 (DHR123) (MedChemExpress, Cat# HY‐101894). Cells were incubated with these antibodies for 40 min at room temperature in the dark, followed by two washes with cell staining buffer to remove unbound antibodies. For intracellular staining, cells were first fixed and permeabilized using the BD Cytofix/Cytoperm Fixation/Permeabilization Kit (BD Biosciences, Cat# 554 714) following the manufacturer's protocol. Cells were then incubated in 1× BD Perm/Wash Buffer with the following antibodies: RIPK1 (PE‐conjugated, Cell Signaling Technology, Cat# 14 577), SFTPC (Thermo Fisher, Cat# PA5‐71680). Cells were incubated for 40 min at room temperature in the dark, followed by two washes with 1× BD Perm/Wash Buffer to remove unbound antibodies. For SFTPC staining, an unconjugated primary antibody was used, followed by incubation with a fluorochrome‐conjugated secondary antibody: Alexa Fluor 488‐conjugated anti‐rabbit IgG (Thermo Fisher, Cat# A‐11008). Cells were incubated for 40 min at room temperature in the dark and subsequently washed twice with 1× BD Perm/Wash Buffer. Cell apoptosis was assessed using the Annexin V‐FITC/PI Apoptosis Detection Kit (Abcam, Cat# ab14085) following the manufacturer's protocol. Briefly, cells were collected and washed twice with cold phosphate‐buffered saline (PBS). After centrifugation at 300 ×g for 5 min, the cell pellet was resuspended in 1× Annexin V binding buffer at a density of 1×10^6^ cells/mL. A 100 µL aliquot of the cell suspension was incubated with 5 µL of Annexin V‐FITC and 5 µL of propidium iodide (PI) in the dark at room temperature for 15 min. Following incubation, 400 µL of 1× binding buffer was added to each sample. Stained cells were analyzed using FACSVerse flow cytometry, and data files were processed with FlowJo 10.0.7 software.

### Cell Viability

Cell viability was assessed using the MCE Cell Titer‐Glo® Luminescent Cell Viability Assay Kit (Cat# HY‐K0302). Briefly, cells were seeded in 96‐well white opaque plates at a density of 10000 cells/well in 100 µL culture medium and incubated overnight (37 °C, 5% CO_2_) to allow adherence. Following treatment with experimental compounds, the Cell Titer‐Glo® Reagent was equilibrated to room temperature, and 100 µL of the reagent was added directly to each well. Plates were gently mixed for 2 min on an orbital shaker and incubated at 22–25 °C for 10 min to stabilize the luminescent signal. Luminescence was quantified using a luminometer with an integration time of 1 s/well. Data were normalized to untreated control wells.

### Neutrophil Migration Assay

Chemotaxis was assessed using 24‐well Transwell inserts (8‐µm pore size; Corning, Cat# CLS3464). Conditioned medium (AEC‐CM) from LPS‐treated MLE‐12 cells (with or without *Ripk1* knockdown) was collected, centrifuged at 500 ×g for 5 min, and added to the lower chamber. Bone marrow‐derived neutrophils (5×10^5^ cells/well in serum‐free RPMI) were seeded in the upper chamber and incubated for 4 h at 37 °C. After incubation, non‐migrated cells on the upper side of the membrane were gently removed with a cotton swab. The migrated cells on the underside of the membrane were fixed with 4% paraformaldehyde (PFA) for 15 min, then stained with 0.1% crystal violet (Sigma–Aldrich, Cat# C6158) for 10 min. After rinsing with distilled water and air‐drying, images were obtained using a Keyence microscope.

### Co‐Immunoprecipitation (Co‐IP)

To investigate the interaction between RIPK1 and JAK1, MLE‐12 cells were stimulated with 1 µgmL^−1^ LPS for 24 h before protein extraction. Cells were lysed in ice‐cold IP lysis buffer (Thermo Fisher, Cat# 87 788) supplemented with protease and phosphatase inhibitors (Roche, Cat# 0 469 315 9001 and Cat# 0 490 684 5001, respectively). After incubation on ice for 30 minutes, lysates were clarified by centrifugation at 12000 × g for 15 min at 4 °C, and total protein concentration was determined using a BCA assay (YEASEN, Cat# 20201ES76). For immunoprecipitation, 2000 µg of total protein was incubated overnight at 4 °C with either: Anti‐RIPK1 (Cell Signaling Technology, Cat# 34 934) to pull down RIPK1‐associated proteins, Anti‐JAK1 (Cell Signaling Technology, Cat# 50 996) to reciprocally pull down JAK1‐associated proteins, and Normal rabbit IgG (Cell Signaling Technology, Cat# 3900) as a negative control. The next day, 50 µL of Protein A/G magnetic beads (Thermo Fisher, Cat# 88 803) were added, and samples were incubated for 6 h at 4 °C with gentle rotation. Immunoprecipitates were washed five times with lysis buffer, eluted in 2× Laemmli buffer, and heated at 95 °C for 5 min. Eluted protein complexes and input lysates were resolved by SDS‐PAGE and transferred to PVDF membranes (Millipore, Cat# IPVH00010). Membranes were blocked in 5% BSA for 1 h and incubated overnight at 4 °C with the following primary antibodies: RIPK1 (Cell Signaling Technology, Cat# 34 934), JAK1 (Cell Signaling Technology, Cat# 50 996), JAK2(Abcam, Cat# ab108596), JAK3 (Cell Signaling Technology, Cat# 8863), TYK2(Abcam, Cat# ab303500) and β‐Actin (Proteintech, Cat# 66009‐1‐Ig, loading control for input). Membranes were washed with TBST and incubated with HRP‐conjugated secondary antibodies (Cell Signaling Technology, Cat# 7074 for anti‐rabbit, Cat# 7076 for anti‐mouse) for 1h at room temperature. Signals were visualized using an enhanced chemiluminescence kit (ECL, Epizyme, Cat# SQ201) and visualized with an ImageQuant LAS 4000 imager.

### Western Blot

Tissues or cells were lysed in Medium Strength RIPA Lysis Buffer (Epizyme, Cat# PC102) containing a protease and phosphatase inhibitor cocktail (Roche, Cat# 0 469 315 9001). The protein concentration was determined using the BCA Protein Detection Reagent (YEASEN, Cat# 20201ES76). Protein samples (30 µg) were separated by 10% SDS‐PAGE and transferred to PVDF membranes (Millipore, Cat# IPVH00010). PVDF membranes were blocked with Protein‐Free Rapid Blocking Buffer (Epizyme, Cat# PS108P) at room temperature for 20 min and incubated with primary antibodies at 4 °C overnight. The following primary antibodies were used: anti‐P‐RIPK1 (S166) (Arigo Biolaboratories, Cat# ARG66476), anti‐RIPK1 (Proteintech, Cat# 29932‐1‐AP), anti‐P‐JAK1(Tyr1034/1035) (Cell Signaling Technology, Cat # 33 314), anti‐JAK1 (Abcam, Cat# ab133666), anti‐JAK2 (Abcam, Cat# ab108596), anti‐P‐JAK2(Tyr1007/1008) (Thermo Fisher, Cat# MA5‐42424), anti‐P65 (NF‐κB p65) (Cell Signaling Technology, Cat# 6956), anti‐P‐P65(Phospho‐NF‐κB p65) (Cell Signaling Technology, Cat# 3033), anti‐P38 (p38 MAPK) (Cell Signaling Technology, Cat# 9228), anti‐P‐P38 (Phospho‐p38 MAPK) (Cell Signaling Technology, Cat# 9216S), anti‐ERK1/2 (Cell Signaling Technology, Cat# 9102), anti‐P‐ERK1/2 (Phospho‐ERK1/2) (Thermo Fisher, Cat# MA5‐36265), anti‐JNK (Cell Signaling Technology, Cat# 9252S), anti‐P‐JNK (Phospho‐JNK Tyr185) (Cell Signaling Technology, Cat # 9255), anti‐P‐STAT3 (Tyr705) (Cell Signaling Technology, Cat# 9145), anti‐STAT3 (Proteintech, Cat# 10253‐2‐AP), anti‐RIPK3 (Proteintech, Cat# 17563‐1‐AP), anti‐P‐RIPK3 (T231/S232) (Cell Signaling Technology, Cat# 91 702), anti‐MLKL (Proteintech, Cat# 66675‐1‐Ig), anti‐P‐MLKL (S345) (Cell Signaling Technology, Cat#37 333), anti‐MPO (R&D Systems, Cat# AF3667), anti‐Cleaved Caspase‐3 (Asp175) (Cell Signaling Technology, Cat# 9661), anti‐SFTPC (Abcam, Cat# ab211326) and AQP5 (Abcam, Cat# ab305303).

For normalization, β‐actin (Proteintech, Cat# 66009‐1‐Ig), GAPDH (Proteintech, Cat# 60004‐1‐Ig), and Lamin B1 (Proteintech, Cat# 12987‐1‐AP) were used as loading controls. After primary antibody incubation, membranes were incubated with HRP‐conjugated secondary antibodies (Cell Signaling Technology, Cat# 7076 for anti‐mouse and Cat # 7074 for anti‐rabbit) at room temperature for 1 h. Bands were detected using an enhanced chemiluminescence (ECL) reagent (Epizyme, Cat# SQ201) and visualized with an ImageQuant LAS 4000 imager.

### RNA Extraction and qRT‐PCR

Total RNA was extracted from MLE‐12 cells using the TRIzol reagent (Invitrogen, Cat# 15 596 026) following the manufacturer's instructions. RNA concentration and purity were assessed using a NanoDrop spectrophotometer (Thermo Fisher), ensuring an A260/A280 ratio of 1.8–2.0. RNA integrity was verified by agarose gel electrophoresis. cDNA was synthesized using PrimeScript RT Reagent Kit (Takara Bio, Cat# RR037A) with 1000 ng of total RNA per reaction. Reverse transcription was performed at 37 °C for 15 min, followed by inactivation at 85 °C for 5 s. Quantitative real‐time PCR (qRT‐PCR) was conducted using TB Green® Premix Ex Taq II (Takara Bio, Cat# RR820A) on a QuantStudio 3 Real‐Time PCR System (Thermo Fisher, Cat# A28136). Each reaction was performed in triplicate with a total volume of 20 µL, consisting of 10 µL SYBR Green mix, 1 µL cDNA, 0.4 µM of each primer, and nuclease‐free water. The PCR cycling conditions were as follows: Initial denaturation: 95 °C for 30 s, Amplification (40 cycles): 95 °C for 5 s, 60 °C for 30 s, Melt curve analysis: 65–95 °C in 0.5 °C increments. The *Cxcl1* mRNA expression levels were normalized to β‐Actin as the internal reference gene using the 2^−ΔΔCt^ method.

### Luminex Multiplex Cytokine Assay

Chemokine levels in alveolar epithelial cell supernatants were quantified using the Luminex liquid suspension chip assay with the Bio‐Plex Pro Mouse Chemokine Panel (Bio‐Rad Cat# 12 009 159) following the manufacturer's instructions. Supernatants were collected from MLE‐12 cells transfected with siNC or siRipk1 and stimulated with or without 1 µgmL^−1^ LPS for 24 h. Samples were centrifuged at 10000 × g for 10 min at 4 °C, and 50 µL of the clarified supernatant was used for analysis. Magnetic beads were vortexed, resuspended, and added to a 96‐well plate, followed by incubation with standards, samples, and blank controls at room temperature with 850 rpm shaking for 30 min in the dark. After washing, detection antibodies and Streptavidin‐PE reagent were sequentially added, with incubation steps of 30 and 10 min, respectively. Following a final wash, beads were resuspended and analyzed using the Luminex 200 system (Luminex Corporation, Austin, TX, USA). Fluorescence intensity data were processed using Bio‐Plex Manager software. Standard curves were generated using a 5‐parameter logistic (5‐PL) curve‐fitting model, and cytokine concentrations were calculated.

### CUT&Tag‐qPCR

To investigate the binding of phosphorylated STAT3 (P‐STAT3) to the *Cxcl1* promoter, CUT&Tag‐qPCR was performed using the Vazyme Hyperactive Universal CUT&Tag Assay Kit for qPCR (TD904) following the manufacturer's instructions. MLE‐12 cells were harvested, permeabilized, and incubated overnight at 4 °C with anti‐P‐STAT3 (Cell Signaling Technology, Cat# 9145) or IgG control (Cell Signaling Technology, Cat# 2729). Cells were treated with the pA/G‐Tn5 transposome for 1 h, followed by activation and DNA purification. Purified DNA was analyzed by qPCR using TB Green® Premix Ex Taq (Takara, Cat# RR420A) on a QuantStudio 3 Real‐Time PCR System (Thermo Fisher, Cat# A28136). Data were analyzed using the 2^−ΔΔCt^ method, normalized to IgG controls.

### Dual‐Luciferase Reporter Assay

To investigate the direct functional interaction between STAT3 and the *Cxcl1* promoter, dual‐luciferase reporter assays were performed using the Dual‐Luciferase® Reporter Assay System (Promega, Cat# E1910). The mouse *Cxcl1* promoter region was cloned into the pGL3‐basic luciferase reporter vector (m‐CXCL1‐promoter‐WT‐ZV702). A mutant *Cxcl1* promoter construct (m‐CXCL1‐promoter‐Mut‐ZV702) was generated by site‐directed mutagenesis to alter the predicted STAT3 binding site (from 5′‐AGTTCGGGAAGTTCCCAA‐3′ to 5′‐AGTTCGGACCCCTAAAGGATTCCCAA‐3′). A STAT3 overexpression plasmid (m‐STAT3) and its negative control (m‐STAT3‐NC) were also utilized. MLE‐12 cells were seeded into 24‐well plates at a density of approximately 2×10^4^ cells per well and cultured at 37 °C with 5% CO_2_ until ≈60% confluence. For transfection, 1 µg of *Cxcl1* promoter reporter plasmid (wild‐type or mutant), 1 µg of STAT3 overexpression plasmid or control plasmid, and 100 ng of *Renilla* luciferase internal control plasmid (pRL vector) were co‐transfected into MLE‐12 cells using X‐tremegene HP transfection reagent (ROCHE, Cat# 0 636 623 6001) according to the manufacturer's instructions. Specifically, plasmids and X‐tremegene HP were mixed in 100 µl Opti‐MEM (GIBCO, Cat# 31985‐070), incubated for 20 min at room temperature, and then added to cells in 200 µl Opti‐MEM. After 6 h, the medium was replaced with fresh complete medium containing 10% FBS. Twenty‐four hours post‐transfection, cells were stimulated with 1 µgml^−1^ LPS. Forty‐eight hours after transfection, cells were lysed using 1× Passive Lysis Buffer. Firefly and *Renilla* luciferase activities were measured sequentially using a BioTek Synergy HT luminometer. Firefly luciferase activity was normalized to *Renilla* luciferase activity to control for transfection efficiency. Relative luciferase activity was calculated by dividing the normalized luciferase activity of each experimental group by that of the negative control group (pGL3 basic).

### Isolation of Cytoplasmic and Nuclear Proteins

To isolate cytoplasmic and nuclear proteins from cultured cells, the Nuclear and Cytoplasmic Protein Extraction Kit (Yeasen, Cat# 20126ES50) was utilized, following the manufacturer's protocol. Protease and phosphatase inhibitors (Yeasen InStab Protease Inhibitor Cocktail, EDTA‐free, 100× DMSO Stock Solution, Cat# 20124ES; Yeasen InStab Phosphatase Inhibitor Cocktail, 100× Stock Solution, Cat# 20109ES) were added to all buffers immediately before use to preserve protein integrity. Briefly, cells were harvested and washed with ice‐cold PBS. The cell pellet was resuspended in Cytoplasmic Extraction Buffer A supplemented with the inhibitors, incubated on ice, and centrifuged to separate the cytoplasmic fraction. The remaining pellet was resuspended in Nuclear Extraction Buffer C containing the same inhibitors, incubated on ice with intermittent vortexing, and centrifuged to obtain the nuclear extract.

### Enzyme‐Linked Immunosorbent Assay (ELISA)

To quantify CXCL1 levels in cell culture supernatants, the Mouse CXCL1 ELISA Kit (Elabscience, Cat# E‐EL‐M0014) was used, following the manufacturer's instructions. Briefly, 100 µL of standards or samples were added to each well of a 96‐well plate pre‐coated with an anti‐CXCL1 antibody and incubated for 90 min at 37 °C. After washing, 100 µL of biotinylated detection antibody was added and incubated for 60 min at 37 °C. Following another wash, 100 µL of HRP‐conjugated streptavidin was added and incubated for 30 min at 37 °C. The plate was washed again, and 90 µL of TMB substrate solution was added, followed by a 15‐minute incubation at 37 °C in the dark. The reaction was terminated by adding 50 µL of stop solution, and absorbance was measured at 450 nm using a microplate reader. CXCL1 concentrations were calculated based on a standard curve generated with known concentrations of recombinant CXCL1.

MSD GOLD 96‐well plates (Meso Scale Discovery) were pre‐coated with RIPK1 antibody (BD Biosciences, Cat# 610 458) in 1%BSA in PBS at a final concentration of 0.5µgmL^−1^ for 1h at 37 °C. Plates were washed three times with wash buffer (0.05% Tween 20 in PBS), then blocked in 5% BSA in PBS overnight at 4 °C. Serum samples were incubated with pRIPK1‐S166 antibody (Biolynx, Cat# BX60008) at a final concentration of 0.5µgmL^−1^ for 1h at 37 °C with shaking, and then washed three times with wash buffer. Goat anti‐rabbit SULFO‐tagged detection antibody (Meso Scale Discovery) was diluted 1:500 in 1% BSA in PBS and incubated for 0.5h at 37 °C. After washing three times with wash buffer, 150µL 2x Read Buffer T (Meso Scale Discovery) was added to each well. Plates were quickly analyzed on the MESO QuickPlex SQ 120MM (Meso Scale Discovery).

### Transcriptome Sequencing (RNA‐Seq)

Total RNA was extracted from isolated alveolar epithelial cells using TRIzol reagent (Invitrogen, Cat# 15 596 026) following the manufacturer's instructions. RNA purity and concentration were assessed using a NanoDrop One spectrophotometer (Thermo Fisher), and RNA integrity was evaluated using an Agilent 2100 Bioanalyzer (Agilent Technologies, Cat# G2939BA). Samples with an RNA integrity number (RIN) > 7.0 were considered high‐quality and proceeded to library preparation. RNA sequencing libraries were constructed using the NEBNext® Ultra II RNA Library Prep Kit for Illumina® (New England Biolabs, Cat# E7775L) according to the manufacturer's instructions. mRNA was enriched using poly(A) selection, followed by fragmentation, cDNA synthesis, adapter ligation, and PCR amplification. The final libraries were quantified using a Qubit four Fluorometer (Thermo Fisher, Cat# Q33226) and assessed for size distribution and integrity using an Agilent 2100 Bioanalyzer (Agilent Technologies). Sequencing was performed on an Illumina NovaSeq 6000 platform (Illumina; Cat# 20 012 850) in paired‐end mode (150 bp reads), generating at least 20 million reads per sample. Raw sequencing reads underwent quality control using FastQC (v0.11.9). Adapter sequences and low‐quality bases were trimmed using Trim Galore (v0.6.6). High‐quality reads were aligned to the mouse reference genome (GRCm39) using HISAT2 (v2.2.1). Gene expression quantification was performed with featureCounts (v2.0.1), and differentially expressed genes (DEGs) were identified using DESeq2 (v1.34.0), applying an adjusted p‐value < 0.05 and |log2FC| > 1 as thresholds. Functional annotation and pathway enrichment analyses were conducted using Xiantao Academic (https://www.xiantao.love/) for Gene Ontology (GO) and Kyoto Encyclopedia of Genes and Genomes (KEGG) pathway analysis.

### Semi‐Quantitative Cytokine and Phosphorylation Array

To assess JAK/STAT pathway activation in MLE‐12 cells and inflammatory cytokine expression in serum samples, semi‐quantitative protein arrays were performed using the RayBiotech JAK/STAT Pathway Phosphorylation Array (Cat# AAM‐JAKSTAT‐1, RayBiotech) for Figure 4C and the RayBiotech Mouse Inflammation Antibody Array C1 (Cat# AAM‐INF‐1‐8, RayBiotech) for Figure 5G, following the manufacturer's instructions.

For Figure 4C, MLE‐12 cells were harvested, washed with cold PBS, and lysed in RayBio Lysis Buffer (Cat# AA‐LYS‐1‐8, RayBiotech) supplemented with protease and phosphatase inhibitors. Lysates were clarified by centrifugation at 12000 × g for 15 min at 4 °C, and protein concentrations were determined using a BCA assay (YEASEN, Cat# 20201ES76). 1000 µg of total protein per sample was incubated with the array membranes, which were blocked for 30 min at room temperature, followed by overnight incubation at 4 °C with gentle shaking. After washing, membranes were sequentially incubated with biotinylated detection antibodies for 2h and HRP‐conjugated streptavidin for 1 h at room temperature. Phosphorylation levels were detected using an ECL detection system. For Figure 5G, inflammatory cytokine levels in serum samples were measured using the RayBiotech Mouse Inflammation Antibody Array C1 (Cat# AAM‐INF‐1‐8, RayBiotech). Serum samples were clarified by centrifugation at 10000 × g for 10 min at 4 °C, and 500 µL of each sample was applied to the cytokine array membrane. After blocking for 30 min, samples were incubated overnight at 4 °C with gentle shaking, followed by sequential incubation with biotinylated detection antibodies for 2 h and HRP‐conjugated streptavidin for 1 h at room temperature. Cytokine expression levels were detected using an ECL detection system, and signal intensities were quantified.

### Statistical Analysis

All experimental data were analyzed with strict adherence to randomization and blinding protocols to ensure unbiased results. Animals were allocated to experimental groups using stratified block randomization based on baseline weight and experimental endpoints, with sample processing order randomized across groups to eliminate batch effects. Investigators remained blinded to group assignments during data collection and analysis. Data from the replication experiments in this study were expressed as the mean ± standard deviation. All experiments were independently repeated at least three times. Statistical analyses were performed using GraphPad Prism 9.4.1 software. Comparisons between two independent groups were conducted using Student's *t* test (or Mann‐Whitney U test for non‐normal data). One‐way ANOVA was used to compare means among multiple independent groups. Two‐way ANOVA was performed to analyze the effects of time and operation, followed by Bonferroni's post‐hoc test for multiple comparisons. Survival analysis was conducted using the Kaplan–Meier method and compared with the log‐rank test. A p‐value of less than 0.05 was considered statistically significant.

## Conflict of Interest

The authors declare no conflict of interest.

## Author Contributions

H. S., X. L., and S. G. contributed equally to this work Conceptualization is performed by HS, XL, SG, and WC; Methodology is performed by HS, XL, SG, YJ, XW, ZC, HW, QH, RZ, HX, and LT; Investigation is done by HS, XL, SG, YJ, XW, ZC, HW, and QH; Formal Analysis is done by HS, XL, SG, RZ, HX, and RB; Validation is provided by YJ, XW, ZC, HW, QH, and CM; Resources are provided by RZ, HX; Writing – Original Draft is done by HS, XL, and SG; Writing – Review & Editing is performed by YJ, XW, ZC, HW, QH, RB, CM, LT, YL, JW, and WC; Visualization is done by HS, YJ, XW, and Z.C.; Funding Acquisition is provided by W.C., J.W., Y.L., Q.H., and L.T.; Project Administration is performed by L.T., Y.L.; Supervision is done under W.C., J.W., and Y.L.

## Supporting information



Supporting Information

## Data Availability

The data that support the findings of this study are available from the corresponding author upon reasonable request.
