# Supplementary material for: RIPK1 Drives JAK1‐STAT3 Signaling to Promote CXCL1‐Mediated Neutrophil Recruitment in Sepsis‐Induced Lung Injury
Source: Adv Sci (Weinh). 2025 Sep 15;12(45):e07123. doi: 10.1002/advs.202507123 (PMC12677674; doi:10.1002/advs.202507123)
Supplement: Supplementary file 1 — Supporting Information [file ADVS-12-e07123-s001.docx]

**Supplementary Materials for**

**RIPK1 Drives JAK1-STAT3 Signaling to Promote CXCL1-Mediated Neutrophil Recruitment in Sepsis-Induced Lung Injury**

Hao Sun *et al.*

Corresponding author: Wankun Chen, [chenwank@163.com](mailto:chenwank@163.com;); Jun Wang, [jwangf@shmu.edu.cn](mailto:jwangf@shmu.edu.cn;); Ying Li, [liying@sioc.ac.cn](mailto:liying@sioc.ac.cn)

**List of Supplementary Materials:**

Figure S1 to S8

Table S1 to S3


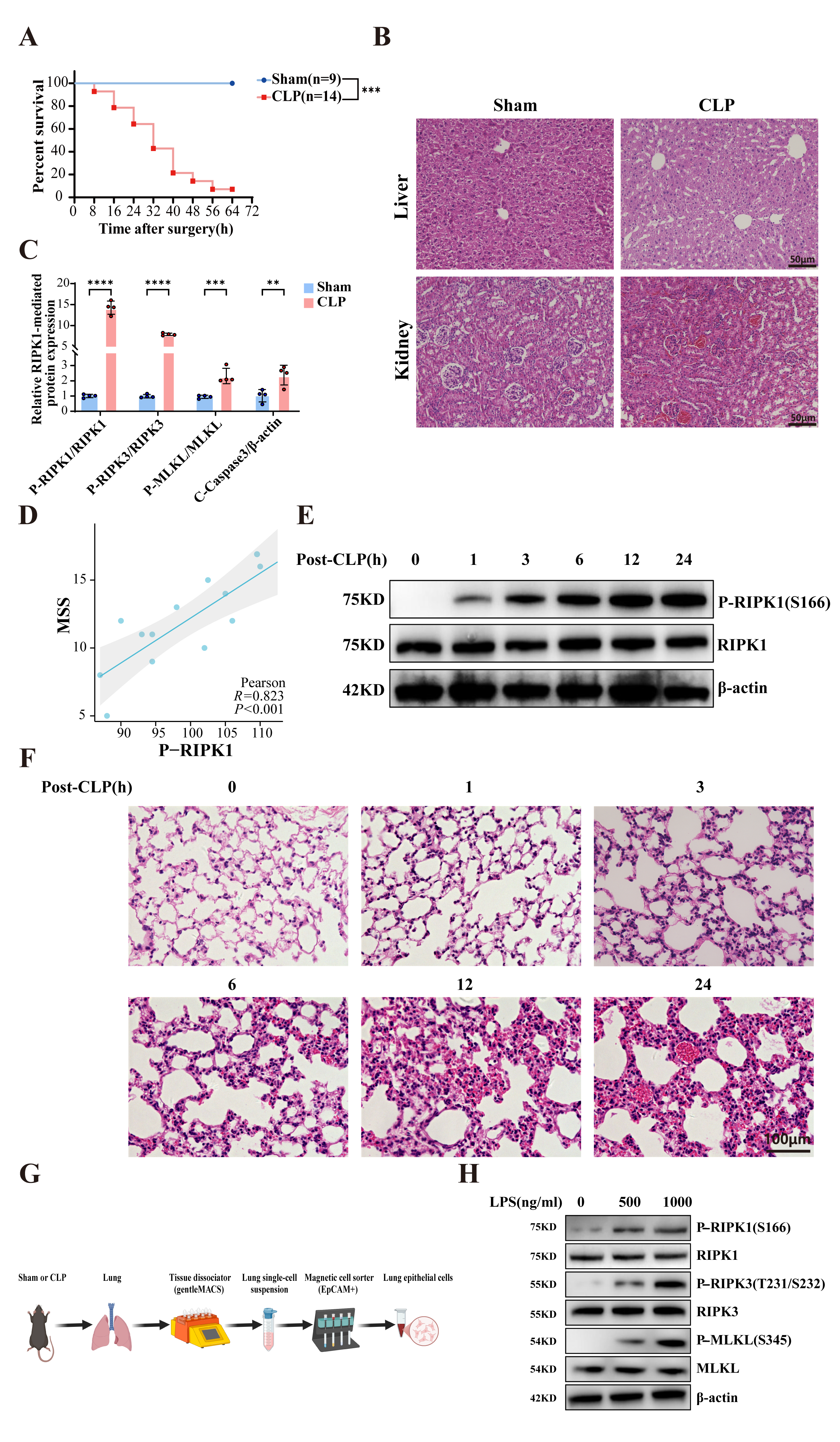


**Figure S1. RIPK1 Activation in CLP-Induced Sepsis and its Distribution in Lung Tissues**

(A) Kaplan-Meier survival curves showing the percentage survival of mice subjected to sham or CLP surgery over 72 hours (Sham, n=9; CLP, n=14). (B) Representative H&E-stained tissue sections of the liver (top panel) and kidney (bottom panel) from sham and CLP groups, demonstrating histopathological changes. Scale bar: 50μm. (C) Quantification of phosphorylated RIPK1 (P-RIPK1 S166), phosphorylated RIPK3 (P-RIPK3 T231/S232), phosphorylated MLKL (P-MLKL S345), and cleaved caspase-3/β-actin in lung tissues from sham and CLP groups (n=4). (D) Correlation between P-RIPK1 expression and modified sepsis severity score (MSS). Pearson’s correlation coefficients and P-values are shown (n=13). (E) Time course of RIPK1 phosphorylation in lung tissue during sepsis. Lung tissues were collected from septic mice at different time points (0, 1, 3, 6, 12, and 24 hours) post-CLP. Western blot analysis was performed to detect the expression of P-RIPK1 (S166), total RIPK1, and β-actin. (F) Time course of histopathological changes in lung tissue during sepsis. Representative H&E staining of lung tissues from septic mice at different time points (0, 1, 3, 6, 12, and 24 hours) post-CLP. Scale bar: 100 μm. (G) Schematic representation of the experimental workflow for isolating lung epithelial cells, including lung dissociation, single-cell suspension preparation, magnetic cell sorting (MACS), and cell validation. (H) Western blot analysis of lung epithelial cells treated with LPS at concentrations of 0, 500, and 1000 ng/mL, showing expression levels of P-RIPK1 (S166), total RIPK1, P-RIPK3 (T231/S232), total RIPK3, P-MLKL (S345), and total MLKL, with β-actin as a loading control. Data are presented as mean ± SD. Survival comparisons were analyzed using the log-rank test (A). Multiple comparisons were analyzed using one-way ANOVA (C). *****P* < 0.0001;****P* < 0.001; ***P* < 0.01; SD, standard deviation; ANOVA, analysis of variance.


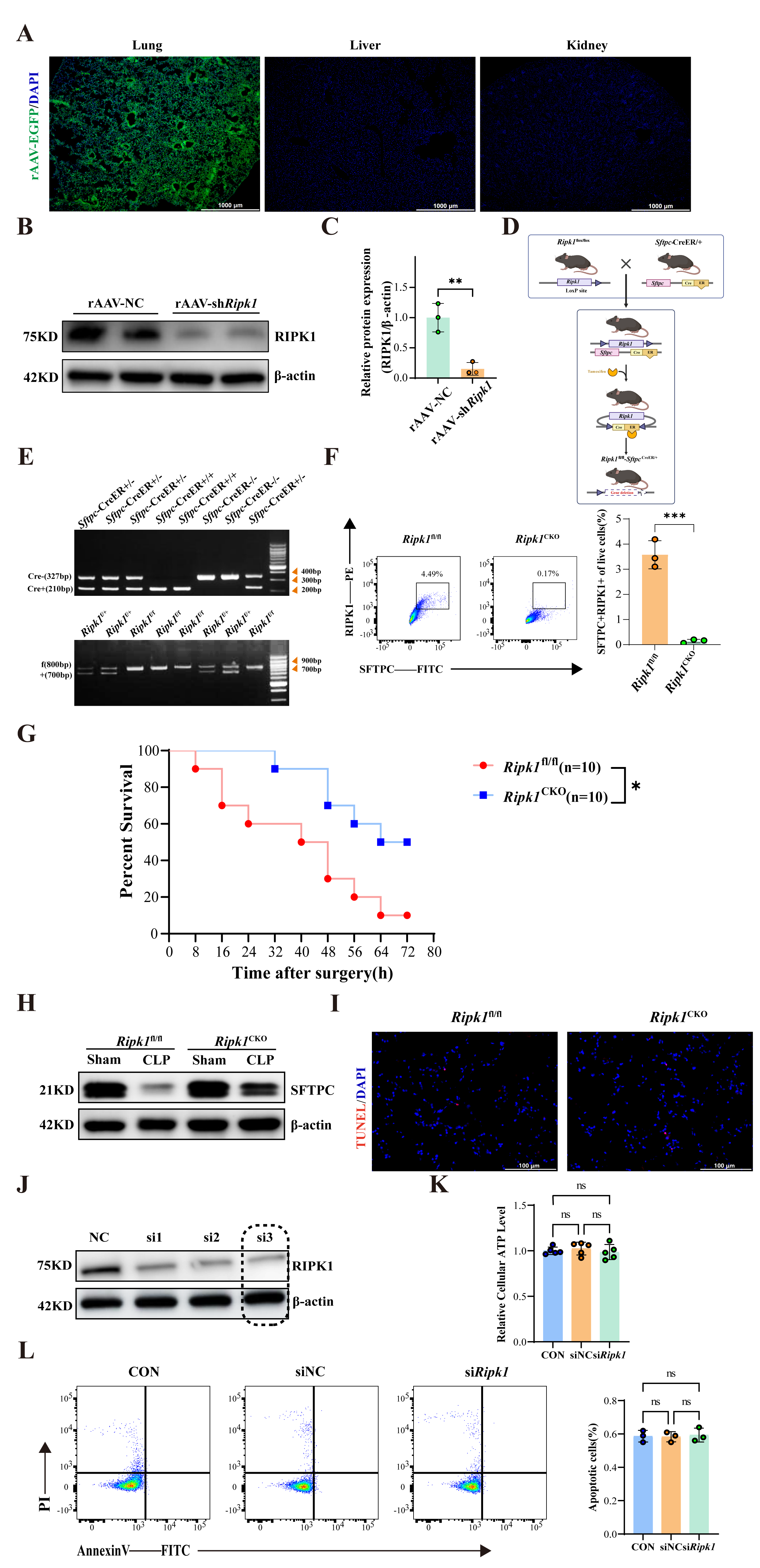


**Figure S2. Lung Epithelial Cell-Specific Role of RIPK1 in Sepsis-Induced Lung Injury**

(A) Representative fluorescence images showing the tissue-specific delivery of rAAV-shRNA targeting RIPK1, with EGFP expression detected in the lung, liver, and kidney tissues. Strong EGFP signals in the lung, while minimal expression is observed in the liver and kidney. Scale bars: 1000μm. (B) Western blot analysis of RIPK1 expression in lung tissue after rAAV-mediated shRNA knockdown. β-actin served as a loading control. (C) Quantification of relative protein expression of RIPK1 in rAAV-NC and rAAV-sh*Ripk1*-treated lungs. (D) Schematic representation of the generation of lung epithelial-specific RIPK1 knockout mice using *Sftpc*-CreER/+ and *Ripk1*^fl/fl^ mice. (E) Genotyping results confirming successful recombination of Sftpc-CreER/+ and *Ripk1*^fl/fl^ alleles. (F) Flow cytometry analysis showing RIPK1 expression in SFTPC+ cells and quantification of the percentage of P-RIPK1+SFTPC+ cells in *Ripk1*^fl/fl^ and *Ripk1*^CKO^ mice. (G) Kaplan-Meier survival curves showing the percentage survival of *Ripk1*^CKO^ and *Ripk1*^fl/fl^ mice subjected to CLP over 72 hours (n=10/group). (H) Western blot analysis showing the expression of SFTPC in lung tissues of *Ripk1*^fl/fl^ and *Ripk1*^CKO^ mice after sham or CLP treatment. β-actin serves as a loading control. (I) Representative TUNEL staining (red) of lung tissues from *Ripk1*^fl/fl^ and *Ripk1*^CKO^ groups. Nuclei were counterstained with DAPI (blue). Scale bar: 100μm. (J) Western blot analysis showing the knockdown efficiency of RIPK1 in alveolar epithelial cells transfected with control siRNA (NC) or three different siRNAs (si1, si2, si3). β-actin served as a loading control. (K) Quantification of relative cellular ATP levels in alveolar epithelial cells under control (CON), negative control siRNA (siNC), and RIPK1 siRNA (si*Ripk1*) conditions (n=5). (L) Flow cytometry analysis of apoptosis in CON, siNC, and si*Ripk1* groups. Cells were stained with Annexin V-FITC and propidium iodide (PI) to distinguish between viable, early apoptotic, late apoptotic, and necrotic cells. Representative dot plots are shown. The proportion of apoptotic cells (%) in CON, siNC, and si*Ripk1* groups was analyzed based on flow cytometry data (n=3). Data are presented as mean ± SD. Two-group comparisons were analyzed using Student’s t test (C, F). Multi-group comparisons were analyzed using one-way ANOVA (K, L). ****P* < 0.001; ***P* < 0.01; NS, no significant difference; SD, standard deviation; ANOVA, analysis of variance; CKO, Conditional Knockout.


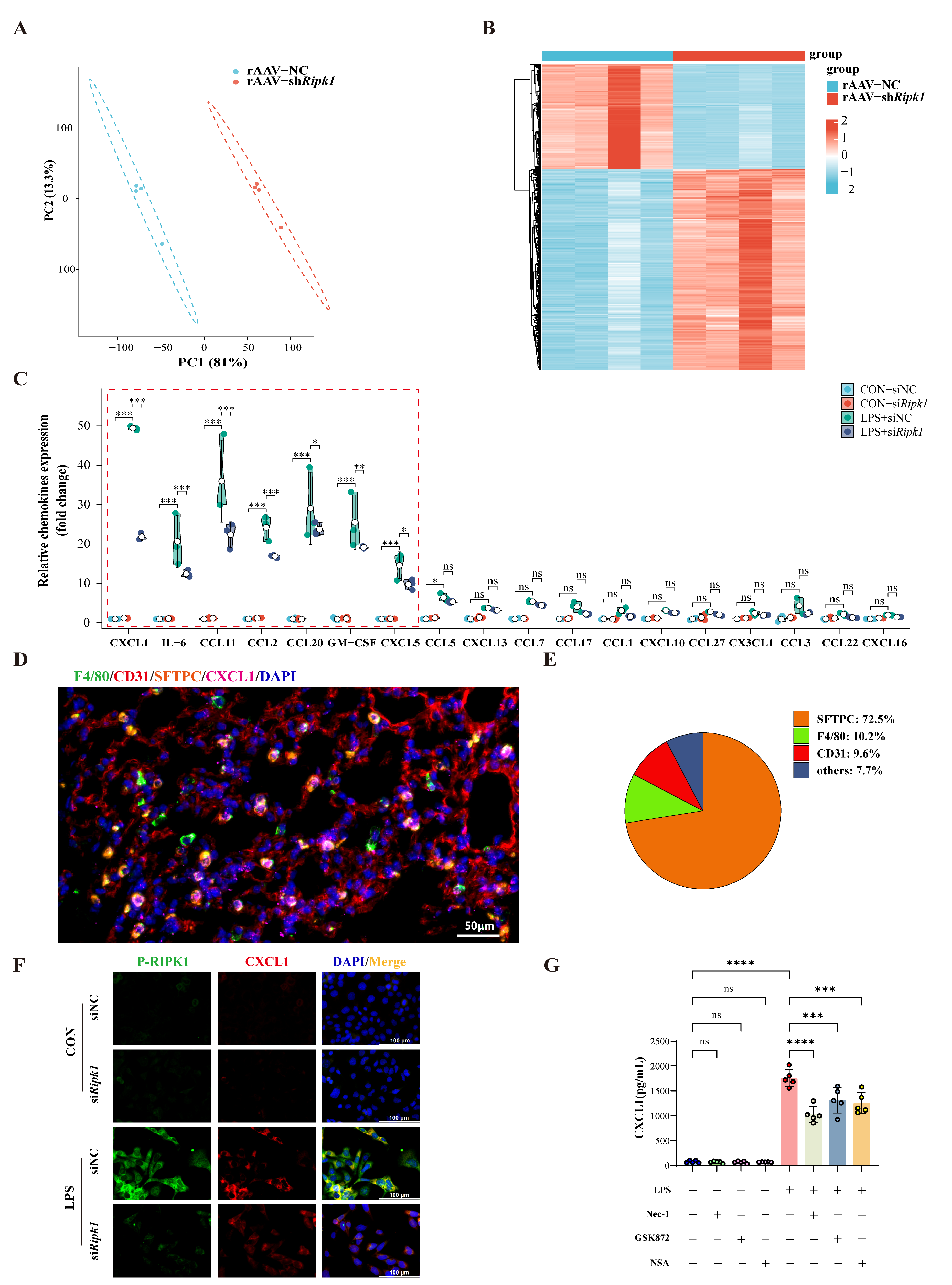


**Figure S3. CXCL1 as a Critical Downstream Chemokine of RIPK1 in Inflammatory Responses**
(A) Principal component analysis (PCA) of gene expression profiles in AECs from rAAV-NC and rAAV-sh*Ripk1* groups, demonstrating distinct clustering between the two groups. (B) Heatmap of differentially expressed genes in lung tissues from rAAV-NC and rAAV-sh*Ripk1* groups, with upregulated genes shown in red and downregulated genes in blue. (C) Quantification of chemokine expression levels in the supernatants of MLE-12 cells under different treatment conditions, measured using Luminex liquid-phase chip technology. Chemokines within the red dashed box are significantly regulated by RIPK1 (n=3). (D) Representative immunofluorescence images of F4/80 (macrophages, green), CD31 (endothelial cells, red), SFTPC (type II alveolar epithelial cells, orange), and CXCL1 (purple), and nuclei (DAPI, blue) in lung tissues from CLP group. Scale bar: 50μm. (E) Pie chart illustrating the percentage of SFTPC-positive, F4/80-positive, CD31-positive, and other cell types in the lung tissue. (F) Representative immunofluorescence images of phosphorylated RIPK1 (P-RIPK1, green), CXCL1 (red), and nuclei (DAPI, blue) in alveolar epithelial cells from control and LPS-treated groups transfected with siNC or si*Ripk1*. Scale bar: 50μm. (G) ELISA analysis of CXCL1 levels in cell culture supernatants under different treatment conditions, including LPS stimulation and co-treatment with inhibitors targeting RIPK1 (Nec-1), RIPK3 (GSK872), and MLKL (NSA) (n=5). Data are presented as mean ± SD. Multiple comparisons were analyzed using two-way ANOVA (C) or one-way ANOVA(G). *****P* < 0.0001; ****P* < 0.001; ***P* < 0.01; **P* < 0.05; SD, standard deviation; ANOVA, analysis of variance.

**
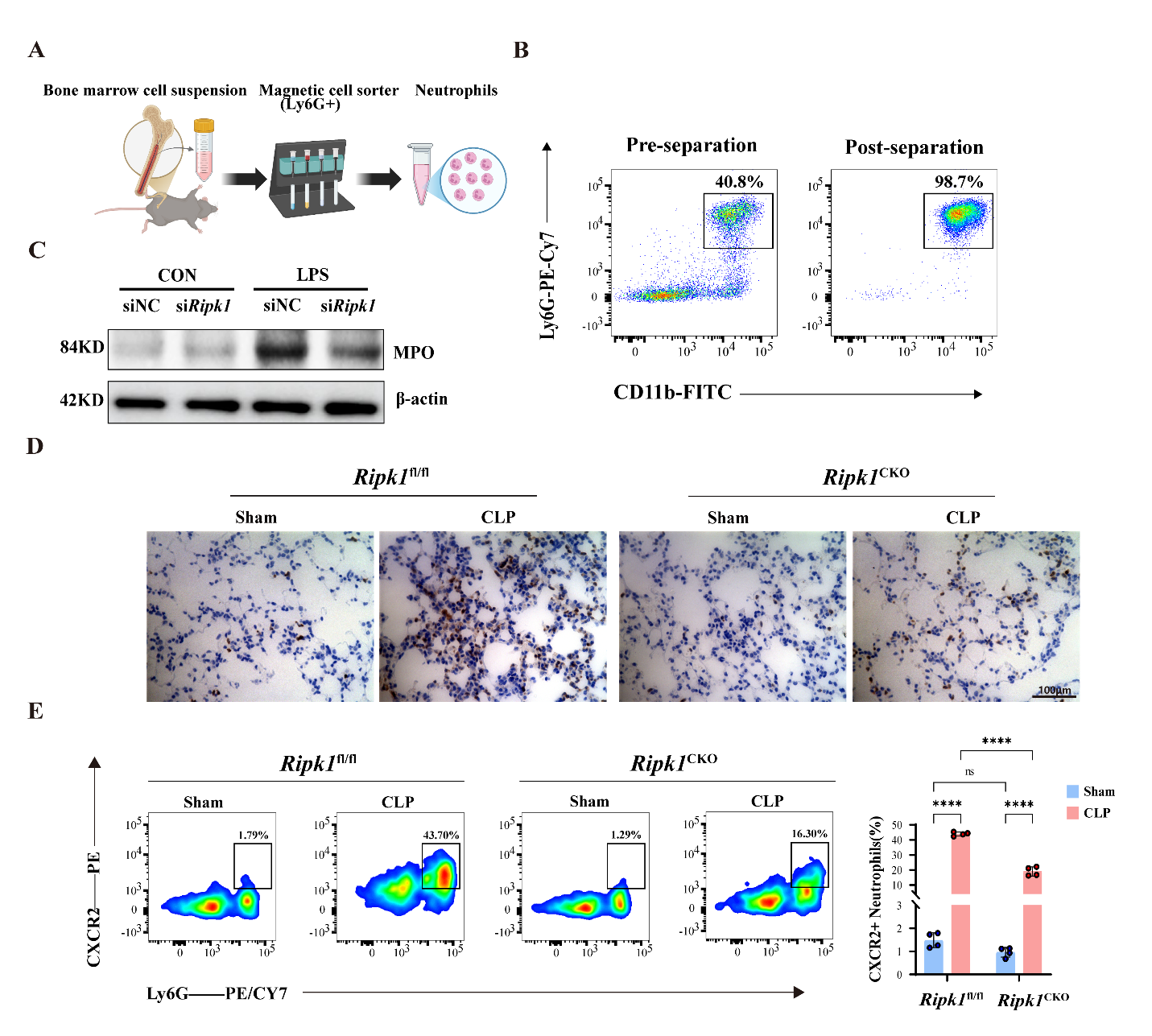
**

**Figure S4. RIPK1 in Alveolar Epithelial Cells Mediates Neutrophil Recruitment in Acute Lung Injury**

(A) Schematic illustration of the workflow for isolating neutrophils from bone marrow using magnetic cell sorting based on Ly6G+ selection. (B) Flow cytometry analysis showing the percentage of neutrophils before (40.8%) and after (98.7%) magnetic sorting, with quantification of neutrophil enrichment. (C) Western blot analysis of MPO expression in neutrophils exposed to alveolar epithelial cell-conditioned medium (AEC-CM). AEC-CM was collected from alveolar epithelial cells treated with control (CON) or LPS and transfected with siNC or si*Ripk1*. β-actin served as a loading control. (D) Representative immunohistochemical staining for Ly6G+ neutrophils in lung tissues of *Ripk1*^fl/fl^ and *Ripk1*^CKO^ mice under sham and CLP conditions. Scale bar: 100μm. (E) Flow cytometry plots showing the percentage of CXCR2⁺ neutrophils in *Ripk1*^fl/fl^ and *Ripk1*^CKO^ mice under sham or CLP conditions. Quantification of CXCR2⁺ neutrophils is shown on the right (n=4). Data are presented as mean ± SD. Multiple comparisons were analyzed using two-way ANOVA (E). *****P* < 0.0001; SD, standard deviation; ANOVA, analysis of variance.


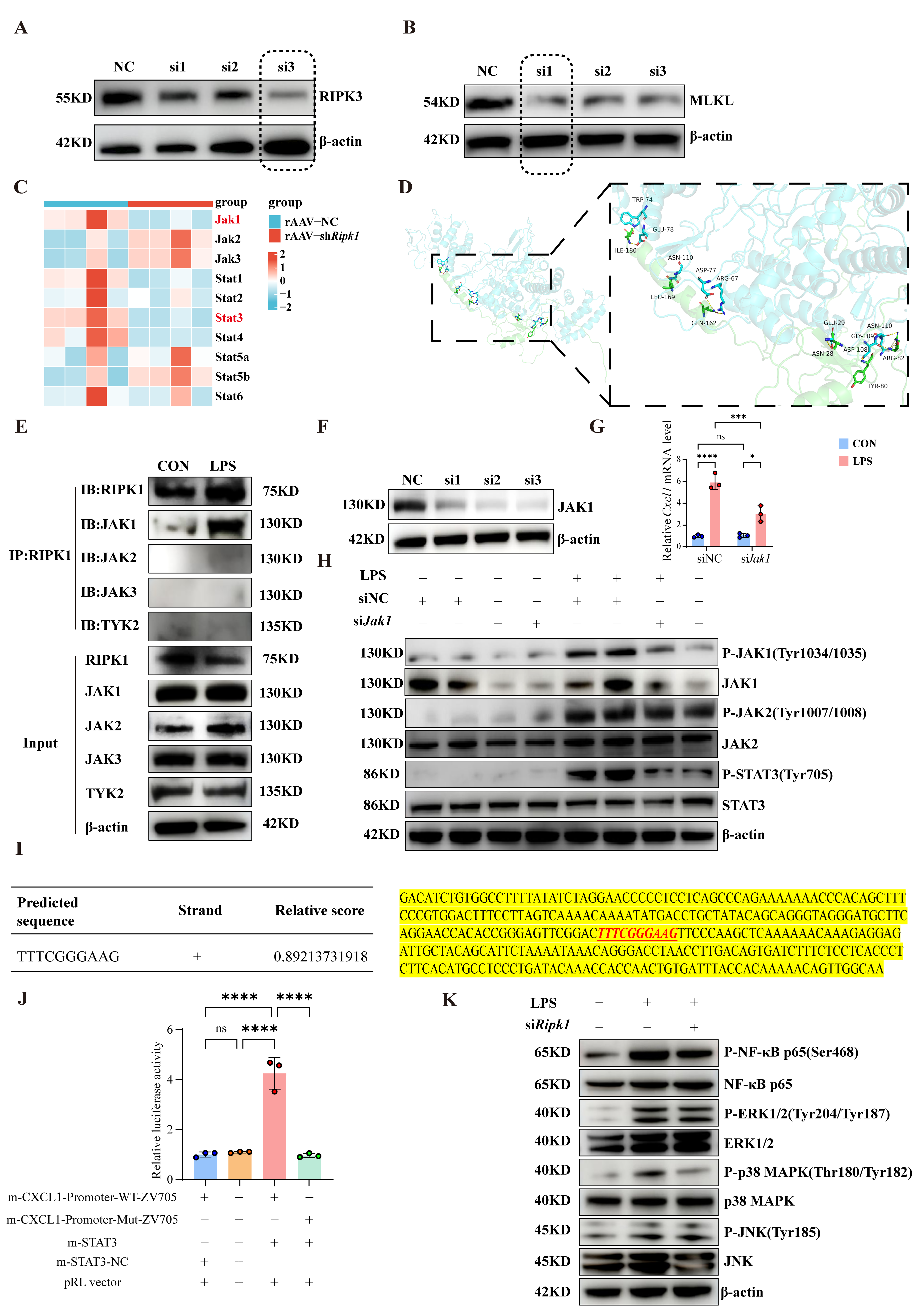


**Figure S5. RIPK1 Promotes *Cxcl1* Transcription Through the JAK1-STAT3 Signaling Pathway**

(A-B) Western blot analysis showing the knockdown efficiency of RIPK3 (A) and MLKL (B) in alveolar epithelial cells transfected with control siRNA (NC) or three different siRNAs (si1, si2, si3). β-actin served as a loading control. (C) Heatmap of differentially expressed genes related to JAK/STAT pathway (Jak1, Jak2, Jak3, Stat1, Stat2, Stat3, Stat4, Stat5a, Stat5b, Stat6) in lung tissues from rAAV-NC and rAAV-sh*Ripk1* groups, with upregulated genes shown in red and downregulated genes in blue. (D) Molecular docking analysis predicting potential binding sites between RIPK1 and JAK1. The enlarged view highlights key interacting residues, including ARG67, ASN110, and GLU78, suggesting potential interfaces for protein-protein interaction. (E) Immunoprecipitation (IP) and Western blot (IB) analysis showing the interaction of RIPK1 with JAK1, JAK2, JAK3, and TYK2, and the input levels of these proteins in control (CON) and LPS-treated groups. (F) Western blot analysis showing the expression levels of JAK1 in cells treated with NC, si1, si2, or si3. β-actin is used as a loading control. (G) Relative mRNA expression levels of *Cxcl1* in cells treated with control (CON) or LPS, with or without siNC or si*Jak1* transfection, as measured by quantitative PCR (n=3). (H) Western blot analysis showing the phosphorylation levels of JAK1 (P-JAK1), JAK2 (P-JAK2), STAT3 (P-STAT3), and total JAK1, JAK2, STAT3, and β-actin in cells under various conditions, including LPS stimulation, and transfection with siNC or si*Jak1*. (I) Identification of a predicted STAT3-binding motif within the *Cxcl1* promoter region using the JASPAR database. The highlighted sequence represents the predicted STAT3-binding site, which was used as the basis for primer design in subsequent experiments. (J) Relative luciferase activity of m-CXCL1-Promoter-WT-ZV705 and m-CXCL1-Promoter-Mut-ZV705 under different conditions, including co-transfection with m-STAT3 or empty vector (n=3). (K) Western blot analysis showing the phosphorylation levels of NF-κB p65 (P-NF-κB p65), ERK1/2 (P-ERK1/2), p38 MAPK (P-p38 MAPK), and JNK (P-JNK), and total NF-κB, ERK1/2, p38 MAPK, JNK, and β-actin in cells treated with LPS or si*Ripk1*. Data are presented as mean ± SD. Multiple comparisons were analyzed using two-way ANOVA (G, J). ****P < 0.0001; ***P < 0.001; *P < 0.05; ns, not significant; SD, standard deviation; ANOVA, analysis of variance.


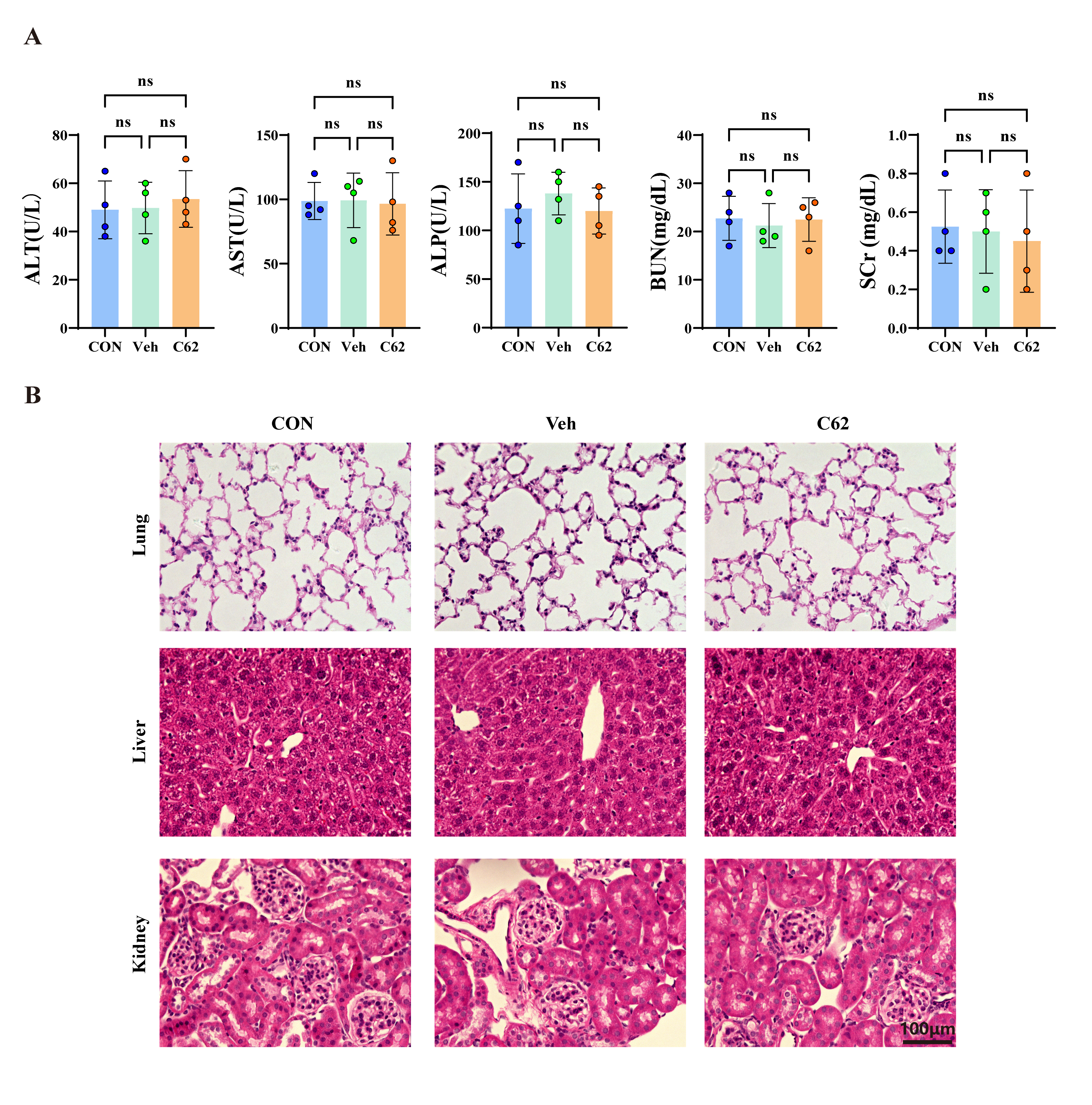


**Figure S6. Safety Evaluation of C62 Administration in Mice**

(A) Analysis of liver and kidney function markers in mice following one week of continuous intragastric gavage administration. Serum levels of Alanine Aminotransferase (ALT), Aspartate Aminotransferase (AST), Alkaline Phosphatase (ALP), Blood Urea Nitrogen (BUN), and Serum Creatinine (SCr) were measured in control (CON), vehicle (Veh), and C62-treated groups (n=4). (B) Representative hematoxylin and eosin (H&E) staining images of lung, liver, and kidney tissues from mice after one week of continuous intragastric gavage administration with control (CON), vehicle (Veh), or C62. No significant pathological changes were observed in any of the organs across the groups, indicating the safety of C62 administration. Scale bar: 100μm. Data are presented as mean ± SD (A). Statistical significance was determined by one-way ANOVA. ns, not significant.

**
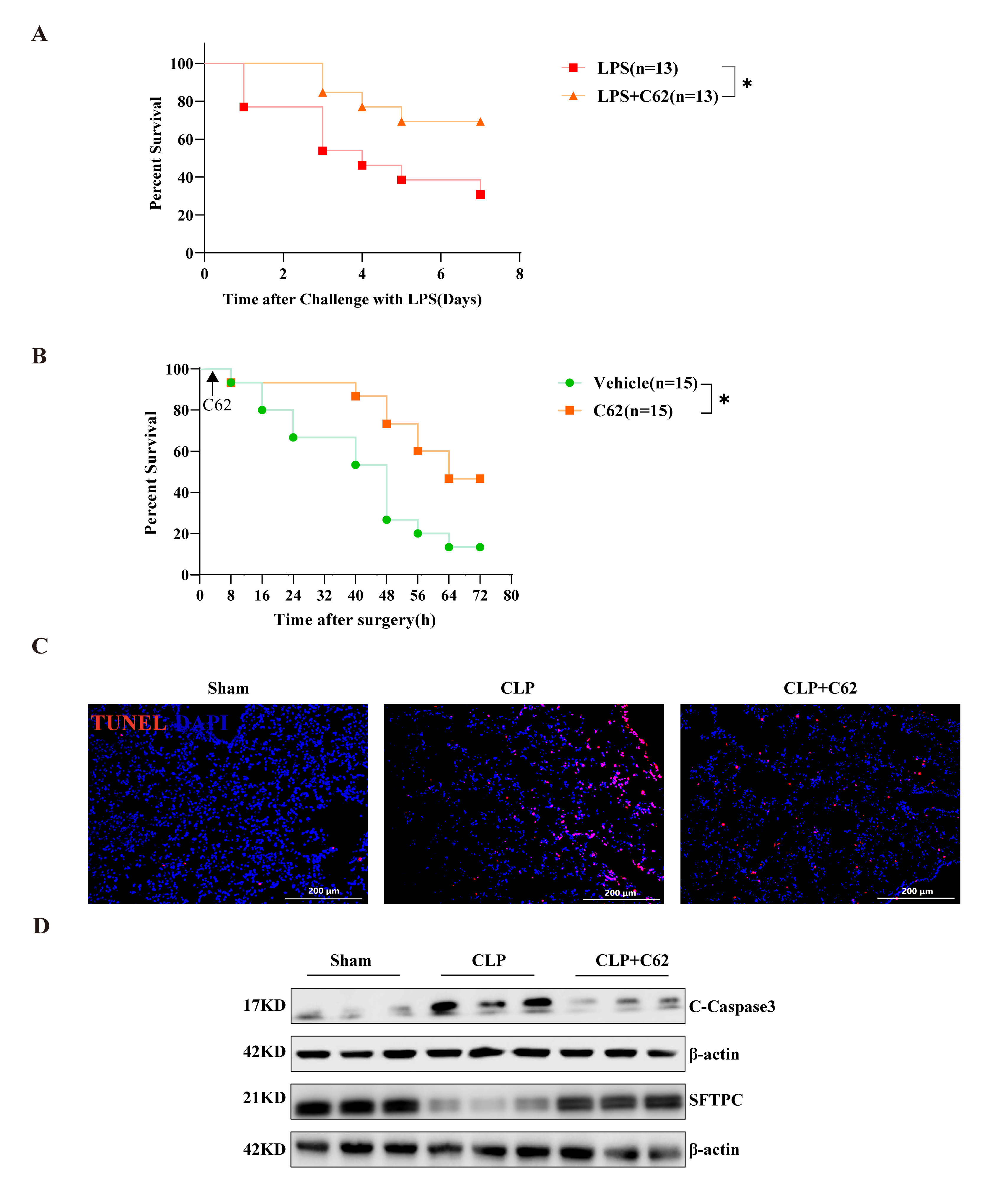
**

**Figure S7. RIPK1 Inhibitor (C62) Improves Survival, and Reduces Apoptosis**

(A) Kaplan-Meier survival curves showing the percentage survival of mice subjected to sham surgery or LPS challenge, with or without RIPK1 inhibitor (C62) treatment (n=13/group). (B) Kaplan-Meier survival curves of mice subjected to CLP surgery. Mice were treated with vehicle (Vehicle group) or C62 (C62 group) (n=15/group). (C) Representative TUNEL staining (red) of lung tissues from sham, CLP, and CLP+C62 groups, showing reduced apoptosis in the CLP+C62 group. Nuclei were counterstained with DAPI (blue). Scale bar: 200μm. (C) Western blot analysis of cleaved caspase-3 (C-Caspase3) and SFTPC in lung tissues from sham, CLP, and CLP+C62 groups. β-actin was used as a loading control. Survival comparisons were analyzed using the log-rank test (A-B). **P* < 0.05.


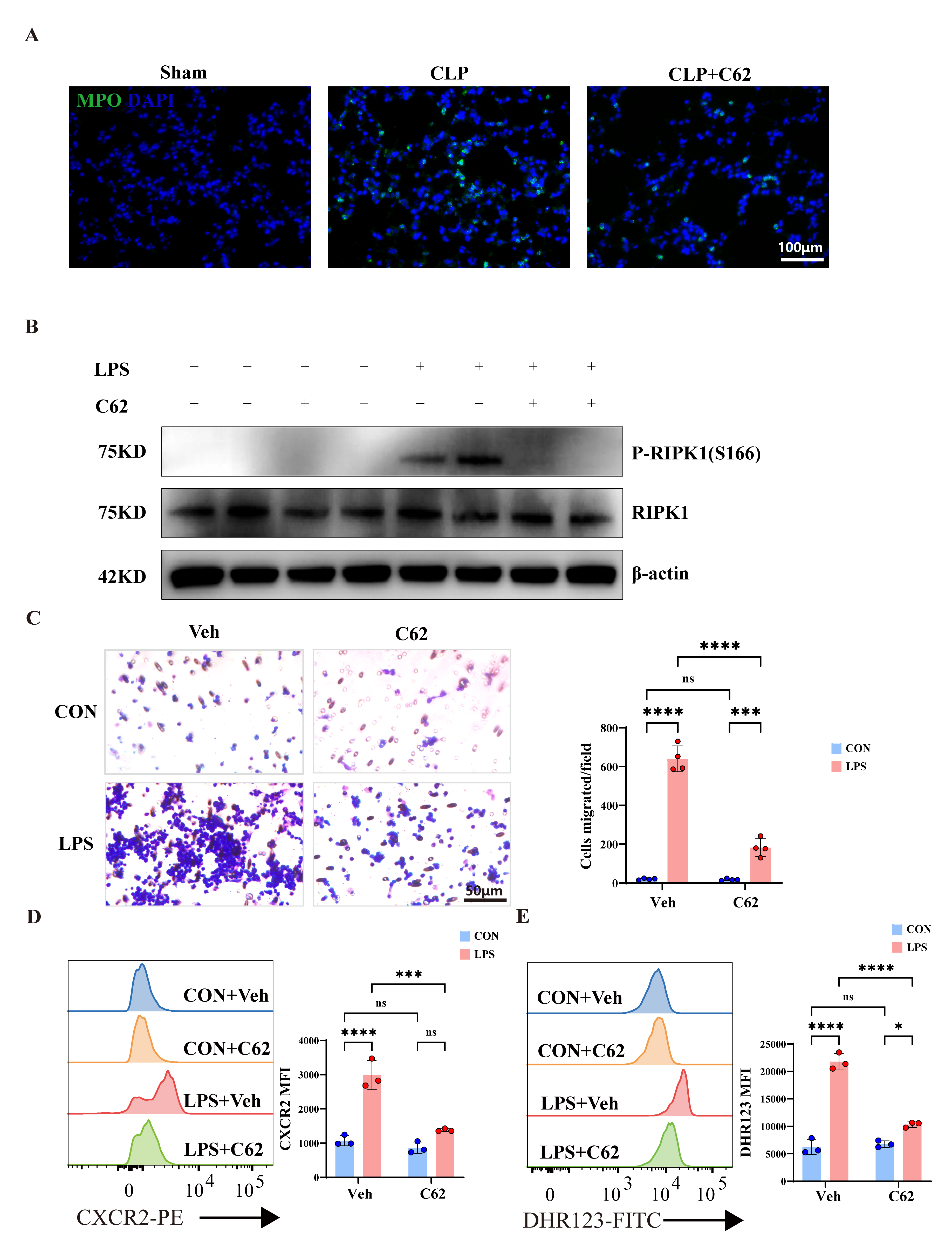


**Figure S8. RIPK1 Inhibitor (C62) regulates inflammatory neutrophil activation**

(A) Representative immunofluorescence images of Myeloperoxidase (MPO, green) and nuclei (DAPI, blue) in lung tissues from Sham, CLP, and CLP+C62 groups, demonstrating neutrophil infiltration. Scale bar: 100μm. (B) Western blot analysis of phosphorylated RIPK1 at Ser166 (P-RIPK1(S166)) and total RIPK1 expression in cells under different treatment conditions, including LPS stimulation and co-treatment with C62. β-actin is shown as a loading control. (C) Quantification of cell migration using a Transwell assay. Representative images of migrated cells are shown for control (CON) and LPS-treated groups co-treated with Vehicle (Veh) or C62. Scale bar: 50μm (n=4). (D) Flow cytometry analysis of CXCR2 expression. Representative histograms and quantification of CXCR2 Mean Fluorescence Intensity (MFI) are shown for CON+Veh, CON+C62, LPS+Veh, and LPS+C62 groups (n=3). (E) Flow cytometry analysis of reactive oxygen species (ROS) production, measured by DHR123-FITC. Representative histograms and quantification of DHR123 Mean Fluorescence Intensity (MFI) are shown for CON+Veh, CON+C62, LPS+Veh, and LPS+C62 groups (n=3). Data are presented as mean ± SD. Multiple comparisons were analyzed using two-way ANOVA(C-E). ****P < 0.0001; ***P < 0.001; *P < 0.05; ns, not significant. SD, standard deviation; ANOVA, analysis of variance.

**Table S1. The clinical profiles of human subjects utilized for experimental validation in this investigation**

|  | Sepsis patients (n = 11) |
| --- | --- |
| Age  Sex, male  SOFA score  Mortality (14d)  *Infection source*  Intra-abdominal sepsis  Meningitis  Urosepsis  Community acquired pneumonia  Intensive care (at point of sampling)  Fluid resuscitation  Nutrition support therapy  Antimicrobial therapy  Ulinastatin  Vasopressors  Blood glucose management  Mechanical ventilation  Renal replacement therapy  White cell count  Proportion neutrophils  Proportion lymphocytes  Proportion monocytes  Proportion eosinophils | 65 (18)  7 (63.6%)  13.4 (2.57)  2 (18%)  3 (27.3%)  0 (0%)  3 (27.3%)  7 (63.6%)  11(100%)  11(100%)  11(100%)  11(100%)  2 (18.2%)  10 (90.9%)  11 (100%)  11 (100%)  1 (9%)  18.5 (9.2)  88%  7%  4%  1% |

Note: Data are n (%) or mean (SD) unless otherwise specified. SOFA=Sequential Organ Failure Assessment on day of sampling.

**Table S2. Chemokines regulated by RIPK1 detected in RNA-seq, Luminex Assay, and Inflammation Array**

| RNA-seq(in vivo) | Luminex assay(in virto) |
| --- | --- |
| CXCL3  CXCL2  CCR3  CSF1  CXCL1  CCL5  CSF2  CCL9  CCR1  CCL20  CXCL5  CXCL10  RAC2 | CXCL1  CCL11  GM-CSF  CCL20  CCL2  IL-6  CXCL5  CCL7 |

**Table S3. Various Types of Primer Sequences**

| **Gene** | **Primer sequence（5’-3’）** |
| --- | --- |
| *Ripk1* siRNA1-F | UAAUGUGAAAGUCACGAUC(dT)(dT) |
| *Ripk1* siRNA1-R  *Ripk1* siRNA2-F  *Ripk1* siRNA2-R  *Ripk1* siRNA3-F  *Ripk1* siRNA3-R  *Ripk3* siRNA1-F  *Ripk3* siRNA1-R  *Ripk3* siRNA2-F  *Ripk3* siRNA2-R  *Ripk3* siRNA3-F  *Ripk3* siRNA3-R  *Mlkl* siRNA1-F  *Mlkl* siRNA1-R  *Mlkl* siRNA2-F  *Mlkl* siRNA2-R  *Mlkl* siRNA3-F  *Mlkl* siRNA3-R  *Jak1* siRNA1-F  *Jak1* siRNA1-R  *Jak1* siRNA2-F  *Jak1* siRNA2-R  *Jak1* siRNA3-F  *Jak1* siRNA3-R  Mouse *Cxcl1* Forward  Mouse *Cxcl1* Reverse  Mouse *Actb* Forward  Mouse *Actb* Reverse  DNA Spike-in-Forward  DNA Spike-in-Reverse  *Cxcl1*-pro-Forward  *Cxcl1*-pro-Reverse | GAUCGUGACUUUCACAUUA(dT)(dT)  UGUAGUUCCAAAUCCAUGC(dT)(dT)  GCAUGGAUUUGGAACUACA(dT)(dT)  UUGAUCUGGAUACUCUUUC(dT)(dT)  GAAAGAGUAUCCAGAUCAA(dT)(dT)  UUAGUGAAGUCUUGUCUAC(dT)(dT)  GUAGACAAGACUUCACUAA(dT)(dT)  UUACCUCGGAGACAGCAGC(dT)(dT)  GCUGCUGUCUCCGAGGUAA(dT)(dT)  UUGUUGAAGACGAGAGCCG(dT)(dT)  CGGCUCUCGUCUUCAACAA(dT)(dT)  UUUAGUGAGCAUUGCUUCAGG(dT)(dT)  CCUGAAGCAAUGCUCACUAAA(dT)(dT)  UGUAGCCUGUAUAAGCCUC(dT)(dT)  GAGGCUUAUACAGGCUACA(dT)(dT)  UUUAGUGCUCUUUGCUGUCCG(dT)(dT)  CGGACAGCAAAGAGCACUAAA(dT)(dT)  GCAUAUUGAUCUCAGAUAATT  UUAUCUGAGAUCAAUAUGCTT  GCUGAAGAGAAGAAGAUAATT  UUAUCUUCUUCUCUUCAGCTT  GCUUGUGAAUACUCUGAAATT  UUUCAGAGUAUUCACAAGCTT  CCAAGTAACGGAGAAAGAAGA  TAGGACCCTCAAAAGAAATTG  CCTCTATGCCAACACAGT  AGCCACCAATCCACACAG  GCCTTCTTCCCATTTCTGATCC  CACGAATCAGCGGTAAAGGT  GGGTAGGGATGCTTCAGGAA  TGAAGAGGGTGAGGAGAAAGATC |
